# Interventions Using Wearable Activity Trackers to Improve Patient Physical Activity and Other Outcomes in Adults Who Are Hospitalized: A Systematic Review and Meta-analysis

**DOI:** 10.1001/jamanetworkopen.2023.18478

**Published:** 2023-06-15

**Authors:** Kimberley Szeto, John Arnold, Ben Singh, Bethany Gower, Catherine E. M. Simpson, Carol Maher

**Affiliations:** 1 Alliance for Research in Exercise, Nutrition and Activity (ARENA), University of South Australia, Adelaide, South Australia, Australia

## Abstract

**Importance:**

Low levels of physical activity during hospitalization are thought to contribute to a range of poor outcomes for patients. Using wearable activity trackers during hospitalization may help improve patient activity, sedentary behavior, and other outcomes.

**Objective:**

To evaluate the association of interventions that use wearable activity trackers during hospitalization with patient physical activity, sedentary behavior, clinical outcomes, and hospital efficiency outcomes.

**Data Sources:**

OVID MEDLINE, CINAHL, Embase, EmCare, PEDro, SportDiscuss, and Scopus databases were searched from inception to March 2022. The Cochrane Central Register for Controlled trials, ClinicalTrials.gov, and World Health Organization Clinical Trials Registry were also searched for registered protocols. No language restrictions were imposed.

**Study Selection:**

Randomized clinical trials and nonrandomized clinical trials of interventions that used wearable activity trackers to increase physical activity or reduce sedentary behavior in adults (aged 18 years or older) who were hospitalized were included.

**Data Extraction and Synthesis:**

Study selection, data extraction, and critical appraisal were conducted in duplicate. Data were pooled for meta-analysis using random-effects models. The Preferred Reporting Items for Systematic Reviews and Meta-analyses guideline was followed.

**Main Outcomes and Measures:**

The primary outcomes were objectively measured physical activity or sedentary behavior. Secondary outcomes included clinical outcomes (eg, physical function, pain, mental health), and hospital efficiency outcomes (eg, length of stay, readmission).

**Results:**

Fifteen studies with a total of 1911 participants were included, representing various surgical cohorts (4 studies), stroke rehabilitation (3 studies), orthopedic rehabilitation (3 studies), mixed rehabilitation (3 studies), and mixed medical (2 studies). All studies were included in meta-analyses. There was a significant association between wearable activity tracker interventions with higher overall physical activity (standardized mean difference, 0.35; 95% CI, 0.15 to 0.54; *I^2^* = 72%; *P* < .002) and less sedentary behavior (mean difference, −35.46 min/d; 95% CI, −57.43 to −13.48 min/d; *I^2^* = 0; *P* = .002), and a significant association between wearable activity tracker interventions with improvements in physical function (standardized mean difference, 0.27; 95% CI, 0.08 to 0.46; *I^2^* = 0; *P* = .006) compared with usual care. There was no significant association between wearable activity tracker interventions with pain, mental health, length of stay, or readmission risk.

**Conclusions and Relevance:**

In this systematic review and meta-analysis, interventions that used wearable activity trackers with patients who are hospitalized were associated with higher physical activity levels, less sedentary behavior, and better physical functioning compared with usual care.

## Key Points

**Question:**

Are interventions that use wearable activity trackers during hospitalization associated with improvements in patients’ physical activity and sedentary behavior and their clinical and hospital efficiency outcomes?

**Findings:**

In this systematic review and meta-analysis of 15 studies and 1911 participants, using wearable activity trackers during hospitalization was associated with higher physical activity, less sedentary behavior, and improved physical function but was not associated with improvements in other clinical or hospital efficiency outcomes.

**Meaning:**

These findings suggest that wearable activity trackers can increase patient physical activity, reduce sedentary behavior, and enhance physical function during hospitalization, which may make them a useful tool for supporting patient recovery.

## Teaser

This systematic review and meta-analysis evaluates the association of wearable activity trackers during hospitalization with patient activity, sedentary behavior, clinical outcomes, and hospital efficiency outcomes.

## Introduction

Periods of hospitalization are characterized by very low levels of patient physical activity (PA).^1,2^ This is often despite patients’ ability to walk independently^3^ and is understood to lead to increased mortality, functional decline, frailty, and disability.^4,5,6^ Additionally, observational studies have shown that higher levels of PA during hospitalization are associated with a shorter length of stay (LOS)^7,8,9,10^ and a reduced rate of readmission.^11,12^ Even small PA volumes of just 900 steps per day during admission appear to prevent functional decline following hospitalization.^13^ The link between low levels of PA during a hospitalization admission and various adverse outcomes suggests that it is critical to address patient PA during a hospital admission.^14^

Efforts to improve patient PA during a hospitalization are growing. Some examples include large-scale policy implementation across more than 40 hospitals in the US supporting changes in patient mobility culture and practices,^15^ early-stage development of PA recommendations for older adults who are hospitalized,^16^ and delivery of a range of interventions targeting patient PA behavior during hospitalization. Behavioral interventions appear effective for increasing PA, but the evidence is inconclusive for physical performance and hospital efficiency outcomes, such as LOS and readmission.^17,18^ Interventions using wearable activity trackers (WATs) are becoming more common in populations who are hospitalized for their ability to promote PA using behavior change techniques, such as self-monitoring, goal setting, and feedback.

WATs are associated with improved PA and health biomarkers,^19^ but their association with hospitalization and patient PA, clinical outcomes (eg, physical function), and hospital efficiency outcomes (eg, LOS) are less understood. This systematic review and meta-analysis aimed to evaluate the association between WATs and patient PA and sedentary behavior (SB) during hospitalization compared with usual care, as well as their association with clinical and efficiency outcomes.

## Methods

This systematic review and meta-analysis was conducted and reported in accordance with the revised Preferred Reporting Items for Systematic Reviews and Meta-analyses (PRISMA) reporting guideline.^20^ We followed a protocol that was registered a priori with PROSPERO (CRD42022315181).

### Data Sources and Search Strategy

We searched OVID MEDLINE, CINAHL, Embase, EmCare, PEDro, SportDiscuss, and Scopus databases from inception to March 2022. Search strategies used keywords and MeSH terms related to patients and hospitalizations, WATs, and PA or SB. The full search strategy is available in eTable 1 in Supplement 1. We screened reference lists of included studies for additional potentially eligible studies, sent a list of included studies to content experts requesting studies that may have been missed, and searched clinical trial registries (ClinicalTrials.gov, Cochrane Central, and World Health Organization Clinical Trials Registry).

### Eligibility Criteria

We analyzed the associations of interventions that used WATs to increase PA or reduce SB among adults who are hospitalized (ie, aged 18 years or older) with medical illnesses, undergoing rehabilitation or surgery, including randomized clinical trials (RCT) and nonrandomized clinical trials (nRCT). Children (ie, aged 18 years or younger), outpatients, patients receiving nonhospital health care services, and studies with single-day or overnight admissions were excluded. Included studies used WATs as the sole intervention or as part of a multicomponent intervention with usual care or no intervention as the control. To be included, studies needed to report on at least 1 objectively measured PA or SB outcome using a WAT (eg, daily step count, minutes of PA or minutes of SB measured by accelerometry), and control groups needed to be blinded to feedback from WATs used for outcome assessment.

### Outcomes

The primary outcomes were objectively measured overall PA (eg, measured as either daily step count, minutes of PA) and SB (eg, daily minutes of SB). Secondary outcomes were specific PA metrics (eg, daily step count, minutes of PA); and hospital efficiency outcomes (eg, LOS, readmission). Physical function refers to a patient’s ability to perform daily activities, such as walking and balancing, as well as their overall physical performance, as measured by various tests, such as walking, mobility, and balance test batteries.

### Study Selection, Data Extraction, and Risk of Bias Assessment

Title and abstract screening and full text review was conducted in duplicate by 2 reviewers (K.S. and either B.S., B.G., or C.S.) using Covidence systematic review software. All discrepancies were resolved by discussion. Data extraction and risk of bias assessment were conducted in duplicate using a custom form to extract data related to study methods, setting, sample demographics and characteristics, intervention and control details, outcome measures, and results. The Joanna Briggs Institute (JBI) critical appraisal checklists for RCTs and for nRCTs were used to assess the risk of bias.^21^ Data and risk of bias discrepancies were resolved by discussion.

### Statistical Analysis

Meta-analysis was performed using RevMan version 5 (Cochrane Community). To evaluate the association between interventions and overall PA, we conducted a meta-analysis by pooling the means and SDs for the main PA outcome from each study. If multiple PA outcomes were available, we used step count because it was reported most frequently and allowed for consistent comparison. Additionally, we conducted separate meta-analyses for SB, and for the secondary outcomes for which sufficient data were available. Mean differences (MDs) with 95% CIs were used to pool continuous outcomes that used the same measurement scale, while standardized mean differences (SMDs) with 95% CIs were used to pool continuous outcomes that used different scales. Count data for readmission were analyzed as dichotomous data by calculating risk ratios. Fixed-effects models were used for meta-analyses of outcomes where there was not considerable heterogeneity, and random-effects models were used for all other meta-analyses due to clinical heterogeneity across studies. Publication bias was assessed using funnel plots where 10 or more studies were included in the meta-analysis,^22^ by plotting SMDs or MDs against corresponding SEs and assessing missing sections or asymmetries. We conducted post-hoc leave-1-out sensitivity analyses for each outcome to evaluate the association of individual studies on the overall results from meta-analyses. Statistical heterogeneity was assessed using a χ^2^ test and* I^2^* statistic. Subgroup analyses by clinical population were performed on overall PA and LOS. If means and SDs were unavailable, we contacted study authors for data; if authors did not respond,^23,24,25,26,27^ we used recommended formulas to convert available data.^22^ For a study with 2 intervention groups and 1 control group,^28^ we used recommended formulas to combine intervention group data to create pairwise comparisons.^22^ Where studies provided results for multiple outcomes for a single construct,^28,29,30^ we prioritized the most comprehensive measure available (eg, physical performance test batteries were prioritized over single-task tests). In 1 study with data available for 2 different mental health constructs^31^ we included each in the meta-analysis and divided sample sizes between comparisons to prevent participants being presented twice.

We assessed the quality of evidence using the Oxford Centre for Evidence-Based Medicine 2011 Levels of Evidence, grading results for each outcome as follows: grade A for consistent level 1 studies (n-of-1 RCTs); grade B for consistent level 2 studies (RCTs) or level 3 studies (nRCTs) or extrapolations from level 1 studies; grade C for level 4 studies (historically controlled studies) or extrapolations from level 2 or 3 studies.^32^ When assigning grades, we considered study quality, precision, directness of results, consistency between studies, and effect sizes.

Two-sided *Z* tests were used to calculate *P* values for the summary effect sizes in the meta-analysis, and the statistical significance level was *P* < .05. Statistical analysis was performed on November 3, 2022, with the most recent update of the analysis performed on April 19, 2023.

## Results

**Figure 1.**
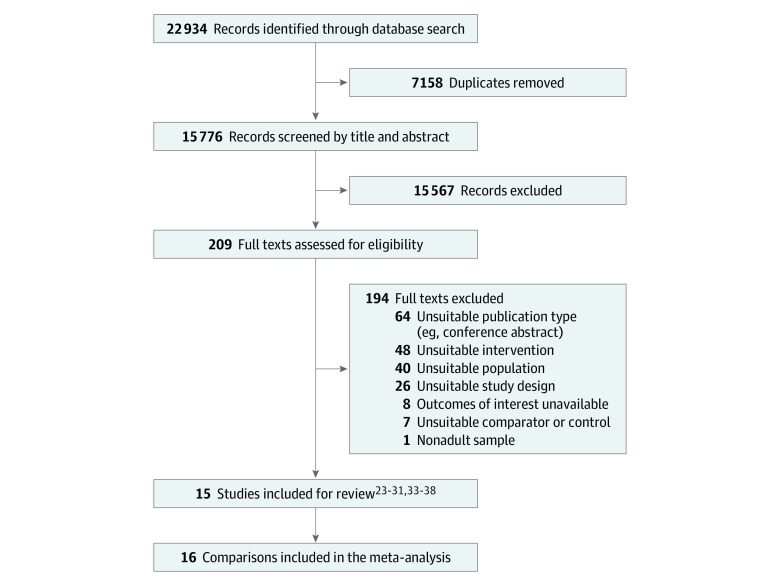
Flow Diagram of Study Selection

We identified 22 934 records from the database search. Following removal of duplicates, 15 776 titles and abstracts were screened. Irrelevant titles and abstracts were excluded, and 209 full texts were screened, with 15 studies^23,24,25,26,27,28,29,30,31,33,34,35,36,37,38^ (11 RCTs and 4 nRCTs) included (Figure 1). A list of studies excluded at full text screening is available in eTable 4 in Supplement 1. All 15 studies were included in the meta-analysis (16 comparisons); 14 studies (15 comparisons) were included in the meta-analysis for overall PA while 2 studies were included in the meta-analysis for SB.

### Study Characteristics

Four studies were conducted in Australia,^29,30,33,36^ 4 in Europe,^24,25,36,37^ 3 in Asia,^27,31,34^ 3 in North America,^26,28,35^ and 1 in the Middle East.^23^ Sample sizes ranged from 41 to 255, with a total sample of 1911 across all studies.^23,24,25,26,27,28,29,30,31,33,34,35,36,37,38^ The mean (SD) age of participants ranged from 52.5 (10.4) to 81 (8) years, and 13 studies^23,24,25,28,29,30,31,33,34,35,36,37,38^ were mixed sex, with more females represented overall (range, 37%-100%). Populations included 4 surgical cohorts,^24,26,27,38^ 3 stroke rehabilitation studies,^28,34,35^ 3 orthopedic rehabilitation studies,^31,36,37^ 3 mixed rehabilitation studies,^29,30,33^ and 2 mixed medical studies.^23,25^ Studies were published between 2013 and 2021, with 12 studies^23,24,25,27,28,29,31,33,34,36,37,38^ published since 2018 (Table).

**Table.** Characteristics of Included Studies

Source	Country	Study design	Sample size (at enrollment)	Population	Age, mean (SD), y	Sex, No. (%)	Intervention summary	Intervention duration	Primary (PA) outcome measures: (1) metric, (2) device used	Secondary outcome measures
Atkins et al,^33^ 2019	Australia	RCT	Total: n = 85; INT: n = 42; CON: n = 43	Mixed/other rehabilitation: mixed rehabilitation with reduced mobility.	Median (IQR): INT, 74 (17); CON, 78 (18).	Female, 46 (59); male, 32 (41)	INT: pedometer intervention plus usual care. Participants recorded steps taken each day as displayed on pedometer. Family and nursing staff encouraged participants to walk more each day and monitor and record their activity. Not tailored to participant. WAT device used: Yamax Digiwalker SW200 pedometer worn on waist band. CON: usual care (individualized exercise prescription).	Hospitalization period only; INT mean (SD), 22 (16); CON mean (SD), 23 (13); range not reported.	1: Daily step count; daily upright time; 2: activPAL accelerometer.	Clinical outcomes: physical function (DMMI, 10-m walk test). Efficiency outcomes: LOS, discharge destination.
Cohen et al,^23^ 2019	Israel	nRCT	Total: n = 377; INT: n = 188; CON: n = 189	Mixed/other medical: mobile older adults (≥65 y) admitted to internal medicine units.	75.4 (7.0), Range: 64-98	Female, 151 (40); male, 226 (60)	INT: multicomponent mobility intervention that used an accelerometer to monitor activity. Unit-tailored mobility program structured for staff, patients, and caregivers, distributed via different methods. Participants prescribed at least 900 steps per day as a goal. Nurses assessed patient mobility and provided recommendations at admission, patient mobility, and walking distance recorded in medical records, unit environment modified to promote patient activity. Not tailored to participant. Intervention based on the System Engineering Initiative for Patient Safety theoretical model. WAT device used: actical (model not described), worn on ankle. CON: usual care (control period prior to intervention implementation)	Hospitalization period only; INT mean (SD), 5.8 (3.0); CON mean (SD), 6.5 (4.3); range not reported.	1: Daily step count; 2: actical accelerometer worn on ankle.	Efficiency outcomes: LOS.
Conijn et al,^24^ 2020	The Netherlands	nRCT	Total: n = 94; INT: n = 52; CON: n = 42	Surgical: elective organ transplantation and vascular surgery.	INT: 57.7 (15.0), CON: 59.1 (13.0)	Female, 41 (44); male, 53 (56)	INT: multicomponent technology-based mobility intervention. Eight digital and nondigital elements: paper and digital information about importance of PA before, during, and after discharge; exercise movie; activity planner; choice of pedometer or accelerometer for participants to monitor their PA; activity coaching with physiotherapist via email or phone; application-based digital exercise program tailored to patient. Uptake of WAT: 64% of participants. Intervention tailored to participant, but WAT component not tailored. WAT device used: participant choice of either Fitbit Flex or Pedometer (make/model not reported), wear location not reported. CON: usual care (physiotherapy based on individual needs and referral by treating physician).	Hospitalization period plus 1 mo postdischarge; hospitalization INT mean (SD), 7.3 (12.2); hospitalization CON mean (SD), 8.3 (10.4); range not reported.	1: Percentage of daily sedentary time; 2: Activ8 Professional Activity Monitor accelerometer worn on anterior thigh.	Efficiency outcomes: LOS.
Dall et al,^25^ 2019	Denmark	RCT	Total: n = 141; INT: n = 72; CON: n = 69	Mixed medical: pulmonary patients (asthma, cancer, COPD, dyspnea, pleural effusion, pneumonia, pneumothorax, other).	INT: 73.8 (12.8); CON: 71.9 (13.6)	Female, 47 (51); male, 46 (49)	INT: daily activity feedback from accelerometer provided to participants via a tablet, presented visually with smiley faces and colors used to represent attainment of target activity levels. Feedback visible to patients, relatives and visitors, and health care staff. Activity targets based on ambulation status at baseline. Activity feedback was tailored based on mobility status. WAT device used: make/model not reported, 2 devices embedded in band aid worn on chest and thigh. CON: Usual care (verbal and written information emphasizing importance of PA)	Hospitalization period only, up to 7 d or until discharge, whichever is shorter.	1: Daily active time (walking and standing); daily sedentary time; 2: two accelerometers (make and model not reported) worn on chest and lateral thigh.	Efficiency outcomes: LOS, 90-d readmission.
Hassett et al,^29^ 2020	Australia	RCT	Total: n = 300; INT: n = 149; CON: n = 151	Mixed/other rehabilitation: adults with mobility limitations undertaking aged care rehabilitation and neurological inpatient rehabilitation.	INT: 70 (18), range: 18-101, CON: 73 (15), range: 21-95	Female, 149 (50); male, 151 (50)	INT: multicomponent digital rehabilitation intervention which included virtual reality, accelerometers for activity monitoring and feedback, and tablet and smartphone applications. Provided 30-60 min sessions for 5 d per week in hospital and postdischarge. Devices provided and activity or exercises prescribed by physiotherapist based on patient goals, mobility limitations, preferences, and digital capabilities. Uptake of WAT: 81% of participants. Intervention tailored to participant, but WAT component not tailored. WAT device used: participant choice of either Fitbit One (worn on pocket, belt, or bra), Fitbit Zip (worn on pocket, belt, or bra), Fitbit Charge (worn on wrist), or Garmin vivofit (worn on wrist). CON: usual care (during hospitalization). Falls prevention brochure and referral to outpatient therapy if required (postdischarge)	6 mo total, beginning at start of hospital admission.	1: Daily step count; daily upright time (active time); 2: activPAL accelerometer.	Clinical outcomes: physical function (SPPB, single leg stance, maximal balance range test, step test); pain (EQ-5D pain or discomfort domain); mental health (SF-6D mental health domain).
Hiraga et al,^31^ 2019	Japan	nRCT	Total: n = 43; INT: n = 21; CON: n = 22	Orthopedic rehabilitation: total knee arthroplasty rehabilitation.	INT: 76.4 (7.1), CON: 76.6 (5.5)	Female, 35 (85); male, 6 (15)	INT: activity diary used for participant to record pedometer-measured steps and daily pain, to facilitate achievement of activity goals and encourage self-management. Focus was emphasized on increasing activity and patient achievement. Goal setting was tailored for participant. WAT device used: Pleasure Walker PZ-150 Pedometer, worn on foot or nonoperative side. CON: usual care (pharmacologic postoperative care, physical therapy (knee joint range of motion and stretching), occupational therapy (goal setting and graded activity specific to goal).	Hospitalization period only, approximately 2-3 wk total; start when occupational therapy services begin (1-2 wk postoperation).	1: Step count; 2: Active Style Pro worn on foot.	Clinical outcomes: pain (NRS), mental health (HADS anxiety, HADS depression). Efficiency outcomes: LOS.
Kanai et al,^34^ 2018	Japan	RCT	Total: n = 55; INT: n = 27; CON: n = 28	Stroke rehabilitation: acute phase ischemic stroke rehabilitation.	INT: 66.8 (10.0), CON: 62.9 (9.1)	Female, 20 (42); male, 28 (58)	INT: participants recorded accelerometer-based physical activity feedback in an exercise calendar. Daily activity targets and long-term goals were set with guidance from therapist, and participants were encouraged to walk 100 to 500 steps more than previous day and feedback was provided in real time via accelerometer. Daily activity targets were modified if participants could not attain original target. Goal setting was tailored to participant. Intervention based on Bandura self-efficacy theory. WAT device used: Fitbit One, worn on waist. CON: usual care (physical activity and rehabilitation program).	Hospitalization period plus 1 mo postdischarge; hospitalization INT mean (SD), 12.2 (2.8); hospitalization CON mean (SD), 11.4 (3.9); range not reported.	1: Daily step count; daily active time (light, moderate, and vigorous); 2: Fitbit One worn on waist.	Efficiency outcomes: LOS.
Klassen et al,^28^ 2020	Canada	RCT	Total: n = 75; INT 1: n = 25; INT 2: n = 25; CON: n = 25	Stroke rehabilitation: inpatient stroke rehabilitation.	57 (11), range: 27-76	Female, 30 (41); male, 44 (59)	INT 1: high dose physical therapy sessions (1 h/d, 5 d/week, for 4 weeks). Targets of completing a minimum of 30 min of activity at an intensity progressing from 40% HRR to >60% HRR, d completing >2000 steps during therapy based off accelerometer feedback. Not tailored to participant. WAT device used: Fitbit One, worn on nonparetic ankle. INT 2: same intervention as INT 1, plus an extra exercise session (1 h/d, 5 d/week, for 4 weeks occurring later in the day). Second session included 30 min weight-bearing walking-related activities and weight-bearing lower extremity exercises (eg, strengthening, balance exercises). Not tailored to participant. WAT device used: Fitbit One, worn on nonparetic ankle. CON: usual care (inpatient physical therapy of progressed upper and lower limb functional exercises as tolerated).	Hospitalization period only, approximately 4 wk total.	1: Step count during exercise sessions; 2: Fitbit One worn on nonparetic ankle.	Clinical outcomes: physical function (6-min walk test, isometric paretic quadricep strength, 5-m walk test); mental health (PHQ-9).
Liebermann et al,^26^ 2013	US	RCT	Total: n = 146; INT: n = 69; CON: n = 77	Surgical: women undergoing major gynecologic surgery.	INT: 56, CON: 53, no SD provided.	Female, 129 (100); male, 0	INT: participants were given an activity goal of at least 500 steps before discharge monitored using a pedometer. Signs were placed around the room to reinforce goal, and health care staff (doctors and nurses) were instructed to remind participants to ambulate. Not tailored to participant. WAT device used: OMRON pedometer, worn around neck. CON: usual care (standard postoperative care with no extra encouragement for ambulation).	Hospitalization period only. Hospitalization INT, mean (no SD), 1.54. Hospitalization CON mean (no SD), 1.71. Range, 1-9 days.	1: Step count in 24 h prior to discharge; 2: OMRON pedometer (model not specified) worn around neck.	Efficiency outcomes: LOS.
Mansfield et al,^35^ 2015	Canada	RCT	Total: n = 60; INT: n = 29; CON: n = 31	Stroke rehabilitation: sub-acute stroke rehabilitation.	Median (min-max): INT: 64 (22-92), CON: 61.5 (24-81)	Female, 21 (37); male, 36 (63)	INT: walking feedback from accelerometer was provided in the context of participants’ own rehabilitation goals and specific and measurable subgoals. Daily reports provided to participants’ physiotherapists, who delivered information based on participants’ goals and presentation. More challenging goals were identified when participants met their subgoals. When participants did not comply with subgoals, alternate strategies for increasing activity into the day were discussed with participants. Goal setting was tailored to participant. WAT device used: Gulf Data Concepts, X6-2mini, 1 worn on each ankle (2 worn total). CON: goal setting and daily physiotherapy, including discussion of progress with therapist without WAT feedback.	Hospitalization period only. Hospitalization INT mean (SD), 14 (13). Hospitalization CON mean (SD), 14 (9). Range, 3-91.	1: Total step count from enrollment until discharge; total active time from enrollment until discharge (walking duration). 2: two Gulf Data Concepts X6-2mini accelerometers, worn around both ankles.	Clinical outcomes: physical function (walking speed [meters per second]). Efficiency outcomes: LOS.
No et al,^27^ 2021	South Korea	RCT	Total: n = 73; INT: n = 37; CON: n = 36	Surgical: midline laparotomy for gynecologic diseases.	INT: 52.5 (10.4), CON: 55.2 (11.9)	Female, 63 (100); male, 0	INT: participants used accelerometer to monitor daily step counts and were encouraged to achieve daily step count goals. Daily activity goals were set as 5%, 15%, 30%, 50%, 80%, 120%, 170%, and 230% of baseline step count. Goal setting was tailored to participant. WAT device used: Lifegram, LA11M-BS, LG, worn on wrist. CON: usual care (did not provide description)	Hospitalization period only 6 d total.	1: Daily step count; 2: LG Lifegram, LA11M-BS accelerometer, worn on wrist.	Clinical outcomes: pain (BPI [mean pain]). Efficiency outcomes: LOS.
Peel et al,^30^ 2016	Australia	RCT	Total: n = 270; INT: n = 135; CON: n = 135	Mixed/other rehabilitation: mixed geriatric rehabilitation (>60 y) including fractures, infections, neurological, cardiopulmonary.	81 (8)	Female, 170 (58); male, 107 (42)	INT: daily walking feedback based on accelerometer data was provided to participants showing walking performed compared with walking targets. Participants set goals and daily walking targets with treating therapist which were reviewed weekly and modified based on accelerometer data to motivate participants to increase activity outside of therapy sessions. All health care staff were trained to use accelerometer data and asked to encourage patients to meet activity goals. Goal setting was tailored to participant. WAT device used: ALIVE Heart and Activity Monitors, Alive Technologies worn on waist, and ActivPal (model not described), worn on mid-thigh. CON: usual care (setting mobility goals, with no feedback from WAT).	Hospitalization period only 4 wk total.	1: Daily active time (walking); 2: ALIVE Heart and Activity Monitors accelerometer worn on waist and activPAL accelerometer worn on midthigh. Changed device part way through due to supply and servicing issues.	Clinical outcomes: physical function (SPPB, gait speed). Efficiency outcomes: LOS, 28-d readmission, discharged to higher level of care.
Van der Walt et al,^36^ 2018	Australia	RCT	Total: n = 202; INT: n = 100; CON: n = 102	Orthopedic rehabilitation: hip or knee arthroplasty rehabilitation.	INT: 67 (9), CON: 66 (9)	Female, 82 (50); male, 81 (50)	INT: participants were given daily step goals that increased during the intervention period, and monitored achievement of this based on step count feedback from accelerometer. Goals were adjusted in circumstances where medical or lifestyle reasons limited activity. Not tailored to participant. WAT device used: Garmin Vivofit 2, worn on wrist. CON: usual care (mobilization commencing on day 1, twice daily physiotherapy sessions for 5 d, inpatient rehabilitation for 7-10 d, outpatient rehabilitation from discharge until 6 wk postoperation)	Hospitalization period plus 4 wk postdischarge, 2 wk hospitalization period.	1: Daily step count; 2: Garmin Vivofit 2 accelerometer, worn on wrist.	Efficiency outcomes: 30-d readmission.
Van Dijk-Huisman et al,^37^ 2020	The Netherlands	nRCT	Total: n = 97; INT: n = 33; CON: n = 64	Orthopedic rehabilitation: total knee arthroplasty or total hip arthroplasty rehabilitation.	Median, INT: 65.10; CON: 66.6	Female, 42 (43); male, 55 (57)	INT: multicomponent digital intervention. Smartphone application linked to accelerometer which provided real-time feedback and clinician-specific and patient-specific information on activity and functional recovery (eg, transfer from the supine position to sitting and vice versa, sit-to-stand, walking). Smartphone application also included a tailored exercise program. Exercise program tailored to participant, but not WAT use. WAT device used: MOX activity monitor, worn on thigh. CON: usual care (twice daily physiotherapy targeting functional recovery and increased PA).	Hospitalization period only hospitalization sample median: 3.	1: Daily active time (standing and walking). 2: MOX accelerometer, worn on thigh.	Efficiency outcomes: LOS.
Wolk et al,^38^ 2019	Germany	RCT	Total: n = 132	Surgical: elective open surgery and laparoscopic surgery of the colon and rectum.	58.9 (11)	Female, 46 (42); male, 64 (58)	INT: participants were given a daily step count target, which they could monitor using an accelerometer. Care staff monitored and provided feedback on patients’ activity data twice daily. Not tailored to the participant. WAT device used: Polar Loop activity tracker, worn on wrist. CON: usual care (did not provide description).	Hospitalization period only 5 d total.	1: Daily step count; cumulative (5-d) active time; 2: Polar Loop activity tracker accelerometer, worn on wrist.	Efficiency outcomes: LOS.

Abbreviations: BPI, Brief Pain Inventory; INT, intervention group; COPD, chronic obstructive pulmonary disease; CON, control group; DMMI, De Morton Mobility Index; EQ-5D, European Quality of Life 5 Dimension; HADS, Hospital Anxiety and Depression Scale; HRR, heart rate reserve; LOS, length of hospital stay; nRCT, nonrandomized clinical trial; NRS, Numeric Rating Scale; PA, physical activity; PHQ-9, Patient Health Questionnaire-9; RCT, randomized clinical trial; SF-6D, Short Form 6 Dimensions Health Survey; SPPB, Short Physical Performance Battery; WAT, wearable device tracker.

### Intervention

Most studies used WATs as the sole intervention, and 4 studies^23,24,29,37^ used WATs in multicomponent interventions. Twelve studies^23,25,26,27,28,31,33,34,35,37,38^ provided the intervention during hospitalization only, with the mean (SD) durations ranging between 1.5 (no SD reported) to 22 (16) days. Three studies^24,29,36^ provided the intervention during hospitalization and postdischarge, with durations ranging between 1 to 6 months. Six studies^25,27,30,31,34,35^ tailored WAT interventions to individuals, 6 studies^23,26,28,33,36,38^ did not tailor interventions, and 3 of the multicomponent interventions tailored some aspects of the intervention but not WAT use.^24,29,37^ Only 2 studies^23,24^ reported that their intervention was based on theory, including Bandura Self-efficacy Theory^34^ and the System Engineering Initiative for Patient Safety theoretical model.^23^ Control groups for all studies received usual care, which varied for different populations (Table).

### Risk of Bias

Eleven studies^25,26,27,28,29,30,33,34,35,36,38^ were assessed with the JBI checklist for RCTs (eTable 2 in Supplement 1), with most performing well in terms of randomization,^26,27,28,29,30,33,34,35,36,38^ allocation concealment,^26,27,28,29,30,33,34,35,36,38^ similar baseline characteristics,^25,26,28,29,30,33,34,35,36,38^ consistent outcome assessment methods,^25,26,27,28,29,30,33,34,35,36,38^ statistical analysis methods,^25,26,27,28,29,30,33,34,35,36,38^ and trial design and conduct.^26,27,28,29,30,33,34,35,36,38^ Domains assessed as high risk for bias included lack of blinding of participants,^25,27,28,29,30,33,35,36,38^ personnel,^25,26,27,28,29,30,33,34,35,36,38^ and assessors^25,26,27,34,36,38^ and not performing intention-to-treat analysis.^25,26,27,28,30,33,34,35,38^ Four studies^23,24,31,37^ were assessed with the JBI checklist for nRCTs (eTable 2 in Supplement 1). All performed well in terms of having a clearly defined intervention, a control group, and statistical analysis methods. Domains assessed as having a high risk for bias were baseline characteristics between groups^23,24,37^ and outcome assessment at multiple time points.^24,37^

### Meta-analysis

#### Overall PA and SB

**Figure 2.**
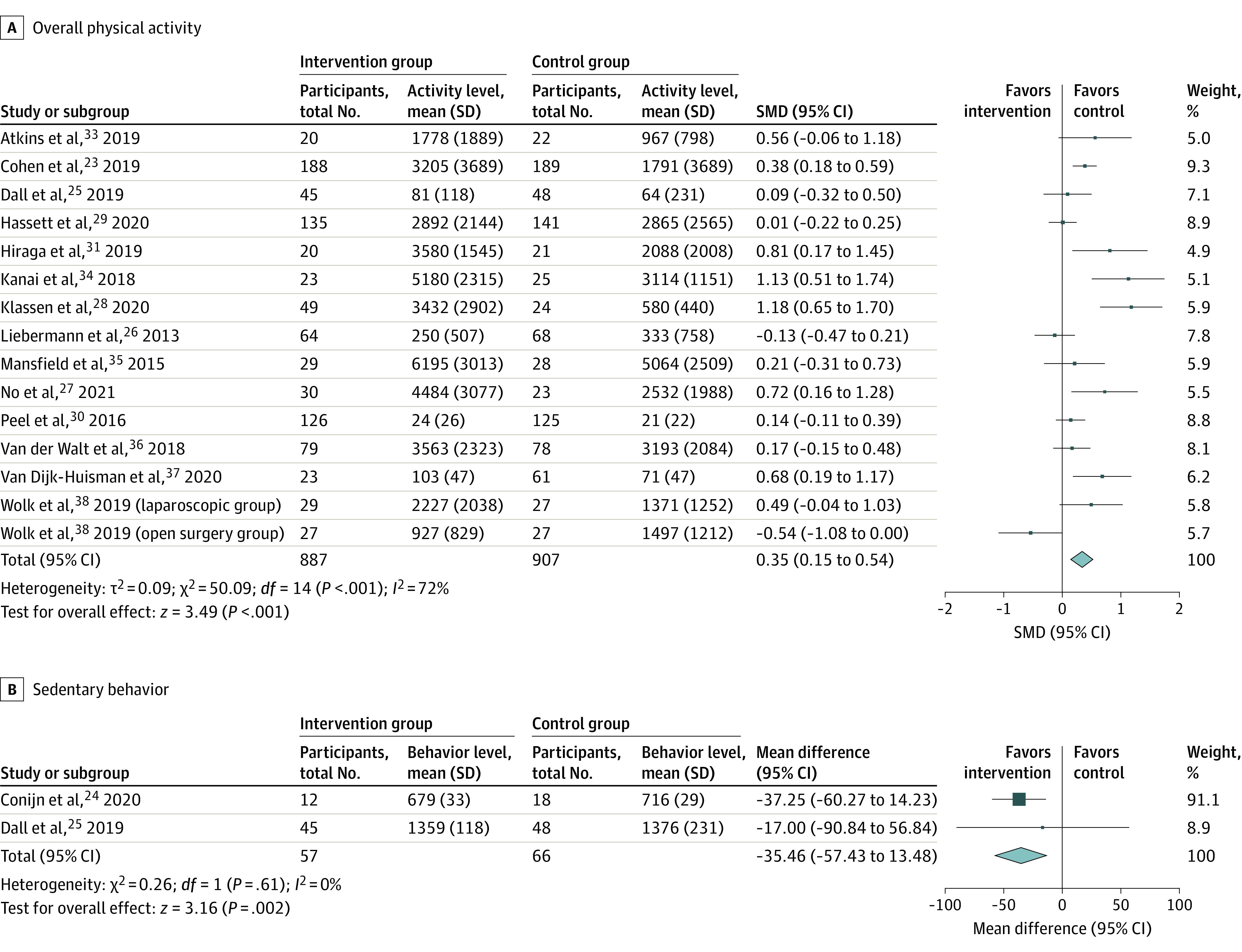
Forest Plot of WAT Intervention Association With Overall Physical Activity and Sedentary Behavior Boxes indicate standardized mean differences (SMDs), with larger boxes reflecting greater weight; horizontal lines indicate 95% CIs; diamonds indicate pooled means, with right and left points indicating 95% CI.

Meta-analysis of 14 studies (15 comparisons)^23,25,26,27,28,29,30,31,33,34,35,36,37,38^ showed WAT interventions were significantly associated with moderately higher overall PA compared with controls with considerable heterogeneity (SMD, 0.35; 95% CI, 0.15-0.54; *I^2^* = 72%; *P* < .001) (Figure 2), which did not differ by clinical subgroup (eFigure 1 in Supplement 1). Two studies^24,25^ reported on SB, with a significant association between WAT interventions and lower minutes of SB, and no heterogeneity (MD, −35.46 min/d; 95% CI, −57.43 to −13.48 min/d; *I^2^* = 0%; *P* = .002) (eFigure 2 in Supplement 1). The level of evidence for WAT interventions association with higher overall PA and lower SB was grade B (level 2 and level 3 studies).

#### Step Count and Active Time

Activity outcomes were available for daily step count (11 studies),^23,26,27,28,29,31,33,34,35,36,38^ and active time (9 studies),^25,29,30,31,33,34,35,37,38^ with 7 studies^25,29,31,33,34,35,38^ reporting more than 1 PA outcome. Data from 11 studies^23,26,27,28,29,31,33,34,35,36,38^ (12 comparisons) showed a significant association between WAT interventions and higher daily step count compared with controls, with high heterogeneity (MD, 826.08 steps/d; 95% CI, 416.92-1235.24 steps/d; *I^2^* = 89%; *P* < .001) (eFigure 2 in Supplement 1). There was a significant association between WAT interventions and higher active time compared with controls, with high heterogeneity (MD, 9.75 min/d; 95% CI, 0.65-18.84 min/d; *I^2^* = 87%; *P* = .04) (eFigure 2 in Supplement 1). The level of evidence for WAT interventions associated with improving each activity outcome was grade B (level 2 and level 3 studies).

#### Clinical Outcomes

**Figure 3.**
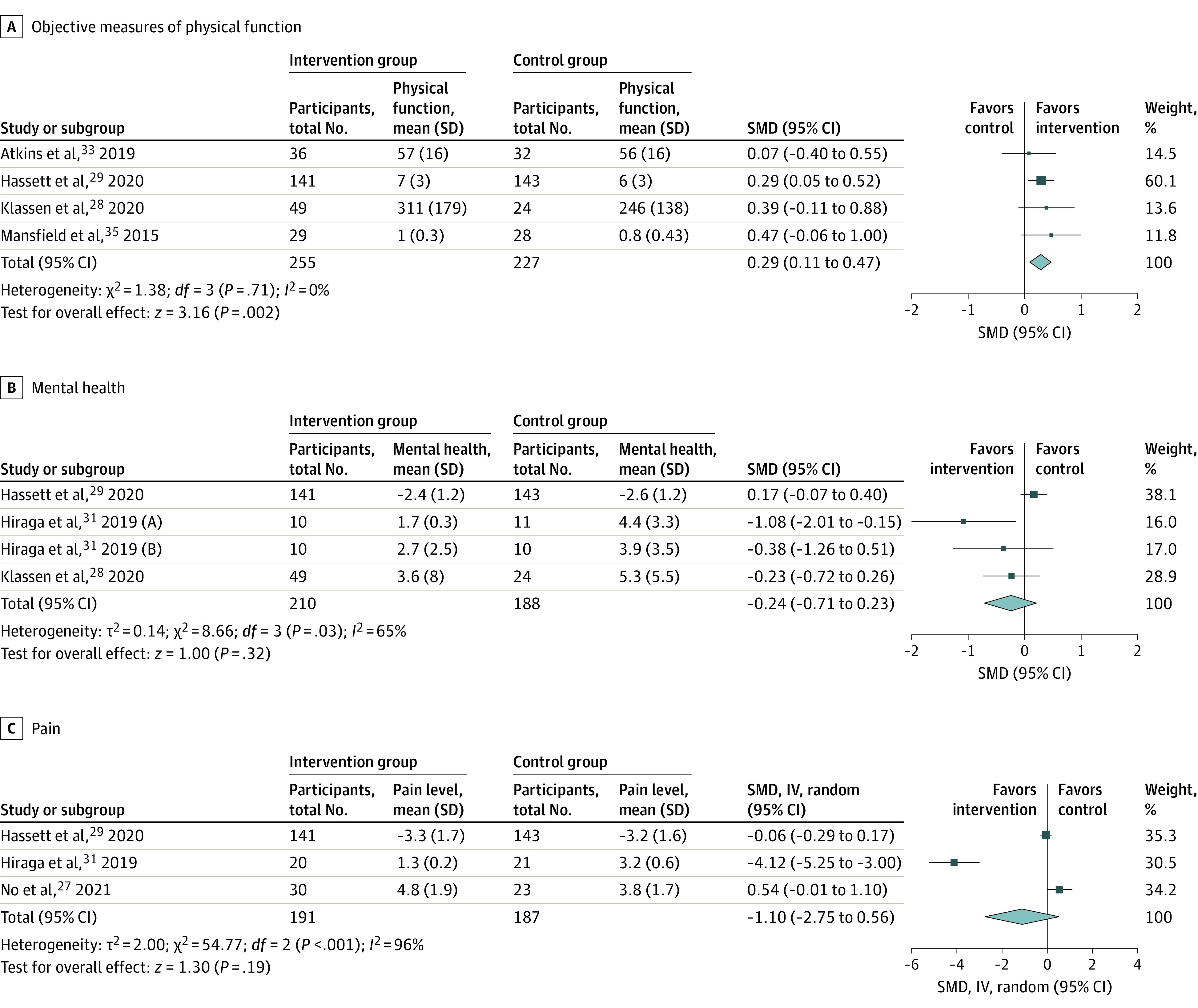
Forest Plot of WAT Intervention Association With Clinical Outcomes Boxes indicate standardized mean differences (SMDs), with larger boxes reflecting greater weight; horizontal lines indicate 95% CIs; diamonds indicate pooled means, with right and left points indicating 95% CI.

Sufficient data for meta-analyses were available for physical function (4 studies),^28,29,33,35^ pain (3 studies),^27,29,31^ and mental health (3 studies; 4 comparisons).^28,29,31^ There was a small, significant association with improvements in physical function favoring WAT interventions compared with control (SMD, 0.27; 95% CI, 0.081-0.46; *I^2^* = 0; *P* = .006) (Figure 3). WAT interventions were found to have a grade B level of evidence for improving physical function, while there was no significant association for pain or mental health outcomes (Figure 3). The level of evidence for WAT interventions association with improving both pain and mental health was grade C due to inconsistency between studies, with a combination of level 2 and level 3 studies.

#### Hospital Efficiency Outcomes

**Figure 4.**
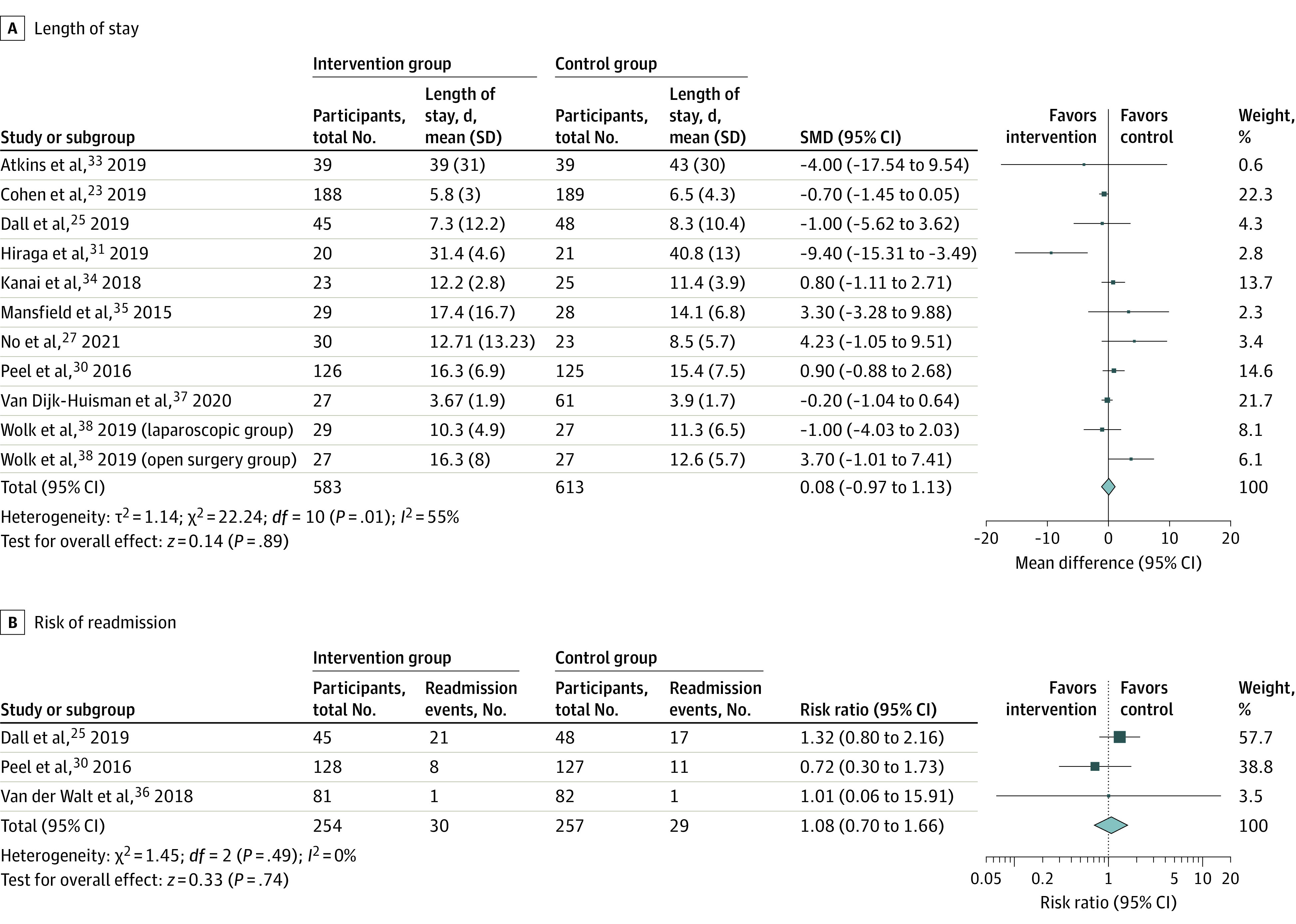
Forest Plot of WAT Intervention Association With Efficiency Outcomes Boxes indicate standardized mean differences (SMDs) and risk ratios, with larger boxes reflecting greater weight; horizontal lines indicate 95% CIs; diamonds indicate pooled means or risk ratios, with right and left points indicating 95% CI.

Ten studies (11 comparisons)^23,25,27,30,31,33,34,35,37,38^ reported on LOS, and 3 studies^25,30,36^ reported on hospital readmission (range, 28-90 days postdischarge). WAT interventions were not significantly associated with LOS or risk of readmission, with moderate heterogeneity (Figure 4). Subgroup analysis by clinical category did not show a significant association with LOS by group (eFigure 1 in Supplement 1). There was no significant association between WAT interventions and risk of readmission (Figure 4). The level of evidence for WAT interventions’ association with improving LOS and risk of readmission was grade C (level 2 and level 3 studies, downgraded due to indirectness of study aims and inconsistency across studies).

### Publication Bias and Sensitivity Analyses

Funnel plots were visually assessed for overall PA, LOS, and daily step count (eFigure 3 in Supplement 1). Plots were symmetrical, indicating no clear evidence of publication bias, and the small number of studies limited exploration of heterogeneity among subgroups. Leave-1-out sensitivity analyses showed consistent associations between WAT interventions and all outcomes, indicating robustness of the key results (eTable 3 in Supplement 1).

## Discussion

Our findings suggest that WAT interventions are associated with significantly higher patient PA and less SB during hospitalization, with potential clinical benefits for patients but not for hospital efficiency outcomes. While a small number of studies showed a significant association between WAT interventions and improved physical function, no improvement was observed in pain and mental health. LOS or readmission showed no significant association with WAT interventions, with limited studies reporting on readmission. However, due to significant heterogeneity among studies, caution is advised when interpreting most of these findings. WAT interventions are a growing area of research, with 80% of the included studies published in 2018 or later.

Our review findings are consistent with broader evidence supporting WAT interventions increasing PA across different populations.^19^ While previous research on WAT interventions in clinical groups has mainly focused on community and outpatient settings, evidence consistently shows improved clinical outcomes, such as aerobic capacity in patients undergoing cardiac rehabilitation^39^ and various cardiometabolic health biomarkers in various chronic diseases.^40,41^ Our findings extends this evidence by suggesting that WAT interventions are also associated with higher PA and improved physical function in populations who are hospitalized. A mean difference of 826 steps per day is substantial, given that increasing daily step counts by even 250 to 500 steps have been associated with reduced risk for adverse hospital outcomes.^42^ Similarly, Agmon et al^13^ identified 900 total daily steps as a threshold for reducing the risk of hospitalization-acquired functional decline; the additional 826 steps per day identified in this study would almost certainly shift patients into the more than 900 steps per day range. While less specific thresholds for active time have been identified, many older adults who are hospitalized only spend 45 minutes per day walking or standing,^3^ so a mean difference of 9.75 active minutes per day appears to be a considerable difference in active time achieved by patients who are hospitalized. This shows promise for using WATs to increase PA and improve patient recovery during hospitalization. As research in this area continues to grow, larger, high-quality trials are needed to strengthen the evidence base.

While the studies included in this review used slightly different definitions and instruments to measure physical function, all instruments included some measure of gait speed as a component. Gait speed is understood to be a useful predictor of survival and disability in older adults,^43^ with increases of just 0.1 m/s associated with a 12% lower risk of mortality at a minimum of 5-year follow-up.^44^ Therefore, even small improvements in physical function associated with WAT interventions during a hospitalization could have substantial benefits for patients. Considering the broader evidence base regarding PA and physical function, it is unsurprising that WAT interventions appear to improve patient physical function during hospitalization. Low levels of PA in patients who are hospitalized are associated with functional decline and increased disability,^4,5,6^ with interventions demonstrating greater improvements in physical function compared to usual care.^45^ Similar associations have been shown in other clinical populations, including hip fracture,^46^ older adults with frailty,^47^ and cancer survivors.^48^ Although pain and mental health showed no significant association, very few studies reported on these outcomes, and effect sizes were favorable. Further research on WAT interventions and clinical outcomes, as well as exploring outcomes in different populations, is warranted. Future work may also extend interventions beyond discharge for sustained impact on PA and other clinical outcomes.

The finding that WAT interventions were not associated with reduced LOS or risk of readmission may seem surprising in light of previous data link higher patient PA with improved hospital efficiency outcomes.^7,8,9,10,11,12^ However, previous associations were found under observational conditions, not experimental conditions. Similarly, the systematic review of Taylor et al^17^ found no association of hospital-based PA interventions on LOS. It is possible that the association between patient PA and LOS or readmission is correlational rather than causal because the decision to discharge a patient is influenced by various factors, including physical function, home and social support, and hospital-specific criteria. Similarly, readmission is also likely to be associated with various factors not addressed by WAT interventions, such as previous hospitalization, medication use and adherence, living arrangements, social support, overall health status, and socioeconomic status.^49,50,51,52^ Further exploration of the economic outcomes associated with WAT interventions in patients who are hospitalized is needed to better understand their costs and benefits.

### Limitations

This study had limitations. The results of our study are limited by the state of the current body of evidence, with only 15 studies identified, most of which involved small samples. Confidence in the overall findings is limited given the small number of included studies. Furthermore, the relatively small evidence base restricted our ability to elucidate the association of variations across studies (eg, populations and intervention characteristics). In particular, limited data were available for clinical outcomes and readmission, with insufficient data for meta-analysis on other clinical and efficiency outcomes (ie, activities of daily living, discharge to higher levels of care). It is possible that more data would reveal additional or different findings.

## Conclusion

This systematic review and meta-analysis found that using WATs during hospitalization is associated with higher patient PA and less SB, along with modest improvements in physical function. Although no association was observed with hospital efficiency outcomes, the growing body of evidence suggests that WATs hold promise for improving patient activity and supporting recovery during hospitalization. As health care becomes increasingly digitized, further exploration of the clinical outcomes and cost-effectiveness of WATs in different groups who are hospitalized will be crucial for guiding their use and maximizing their potential benefits.

## Notes

Supplement 1. **eFigure 1.** Subgroup Meta-Analyses **eFigure 2.** Forest Plot Meta-Analyses for Different Physical Activity Outcomes **eFigure 3.** Funnel Plot Analyses **eTable 1.** Search Strategies **eTable 2. **Risk of Bias **eTable 3.** Leave-1-Out Sensitivity Analyses **eTable 4.** Reason for Exclusion of Studies Screened at Full Text

Supplement 2.
Data Sharing Statement
