# Supplementary material for: Interventions Using Wearable Activity Trackers to Improve Patient Physical Activity and Other Outcomes in Adults Who Are Hospitalized: A Systematic Review and Meta-analysis
Source: JAMA Netw Open. 2023 Jun 15;6(6):e2318478. doi: 10.1001/jamanetworkopen.2023.18478 (PMC10273021; doi:10.1001/jamanetworkopen.2023.18478)
Supplement: Supplement 1. — eFigure 1. Subgroup Meta-Analyses eFigure 2. Forest Plot Meta-Analyses for Different Physical Activity Outcomes eFigure 3. Funnel Plot Analyses eTable 1. Search Strategies eTable 2. Risk of Bias eTable 3. Leave-1-Out Sensitivity Analyses eTable 4. Reason for Exclusion of Studies Screened at Full Text [file jamanetwopen-e2318478-s001.pdf]

Supplemental Online Content

Szeto K, Singh B, Gower B, et al. Interventions using wearable activity trackers to improve patient physical activity and other outcomes in adults who are hospitalized: a systematic review and meta-analysis. *JAMA Netw Open*. 2023;6(6):e2318478. doi:10.1001/jamanetworkopen.2023.18478

eFigure 1. Subgroup Meta-Analyses
eFigure 2. Forest Plot Meta-Analyses for Different Physical Activity Outcomes
eFigure 3. Funnel Plot Analyses
eTable 1. Search Strategies
eTable 2. Risk of Bias
eTable 3. Leave-1-Out Sensitivity Analyses
eTable 4. Reason for Exclusion of Studies Screened at Full Text

This supplemental material has been provided by the authors to give readers additional information about their work.

© 2023 Szeto K et al. *JAMA Network Open.*

eFigure 1. Subgroup Meta-Analyses
eFigure 1.1. Subgroup analysis for WAT intervention association with overall physical activity

Study or Subgroup	Intervention Mean	Intervention SD	Intervention Total	Control Mean	Control SD	Control Total	Weight	Std. Mean Difference IV, Random, 95% CI
**1.1.1 Surgical patients**								
Liebermann et al.[26] 2013	249.82	507.38	64	332.81	757.89	68	7.8%	-0.13 [-0.47, 0.21]
No et al.[27] 2021	4,484.53	3,077.52	30	2,531.97	1,988.09	23	5.5%	0.72 [0.16, 1.28]
Wolk et al.[38] 2018 laparoscopic arm	2,227.2	2,038.4	29	1,371.5	1,251.8	27	5.8%	0.49 [-0.04, 1.03]
Wolk et al.[38] 2018 open surgery arm	927	829.4	27	1,496.6	1,212.33	27	5.7%	-0.54 [-1.08, 0.00]
**Subtotal (95% CI)**			**150**			**145**	**24.8%**	**0.12 [-0.40, 0.64]**
Heterogeneity: Tau² = 0.22; Chi² = 13.74, df = 3 (P = 0.003); I² = 78%								
Test for overall effect: Z = 0.47 (P = 0.64)								
**1.1.2 Stroke rehabilitation**								
Kanai et al.[34] 2018	5,180.5	2,314.9	23	3,113.6	1,150.9	25	5.1%	1.13 [0.51, 1.74]
Klassen et al.[28] 2020	3,431.694	2,901.95318	49	580	440	24	5.9%	1.18 [0.65, 1.70]
Mansfield et al.[35] 2015	6,195	3,013	29	5,604	2,509	28	5.9%	0.21 [-0.31, 0.73]
**Subtotal (95% CI)**			**101**			**77**	**16.8%**	**0.83 [0.19, 1.47]**
Heterogeneity: Tau² = 0.24; Chi² = 7.98, df = 2 (P = 0.02); I² = 75%								
Test for overall effect: Z = 2.55 (P = 0.01)								
**1.1.3 Orthopaedic rehabilitation**								
Hiraga et al.[31] 2019	3,580.5	1,545.2	20	2,088.4	2,008.3	21	4.9%	0.81 [0.17, 1.45]
Van der Walt et al.[36] 2018	3,563.06	2,322.803	79	3,193.49	2,084.13	78	8.1%	0.17 [-0.15, 0.48]
van Dijk-Huisman et al.[37] 2020	102.99	46.757	23	70.896	46.74	61	6.2%	0.68 [0.19, 1.17]
**Subtotal (95% CI)**			**122**			**160**	**19.2%**	**0.49 [0.07, 0.92]**
Heterogeneity: Tau² = 0.08; Chi² = 4.98, df = 2 (P = 0.08); I² = 60%								
Test for overall effect: Z = 2.26 (P = 0.02)								
**1.1.4 Mixed rehabilitation**								
Atkins et al.[33] 2019	1,778	1,888.916757	20	967	797.778	22	5.0%	0.56 [-0.06, 1.18]
Hassett et al.[29] 2020	2,892	2,144	135	2,865	2,565	141	8.9%	0.01 [-0.22, 0.25]
Peel et al.[30] 2016	24	26.1	126	20.6	21.7	125	8.8%	0.14 [-0.11, 0.39]
**Subtotal (95% CI)**			**281**			**288**	**22.8%**	**0.12 [-0.08, 0.33]**
Heterogeneity: Tau² = 0.01; Chi² = 2.75, df = 2 (P = 0.25); I² = 27%								
Test for overall effect: Z = 1.17 (P = 0.24)								
**1.1.5 Medical**								
Cohen et al.[23] 2019	3,205	3,689.06	188	1,791	3,689.06	189	9.3%	0.38 [0.18, 0.59]
Dall et al.[25] 2019	81	118.16	45	64	230.74	48	7.1%	0.09 [-0.32, 0.50]
**Subtotal (95% CI)**			**233**			**237**	**16.4%**	**0.29 [0.03, 0.56]**
Heterogeneity: Tau² = 0.02; Chi² = 1.58, df = 1 (P = 0.21); I² = 37%								
Test for overall effect: Z = 2.17 (P = 0.03)								
**Total (95% CI)**			**887**			**907**	**100.0%**	**0.35 [0.15, 0.54]**
Heterogeneity: Tau² = 0.09; Chi² = 50.09, df = 14 (P < 0.00001); I² = 72%								
Test for overall effect: Z = 3.49 (P = 0.0005)								
Test for subgroup differences: Chi² = 6.18, df = 4 (P = 0.19), I² = 35.3%								

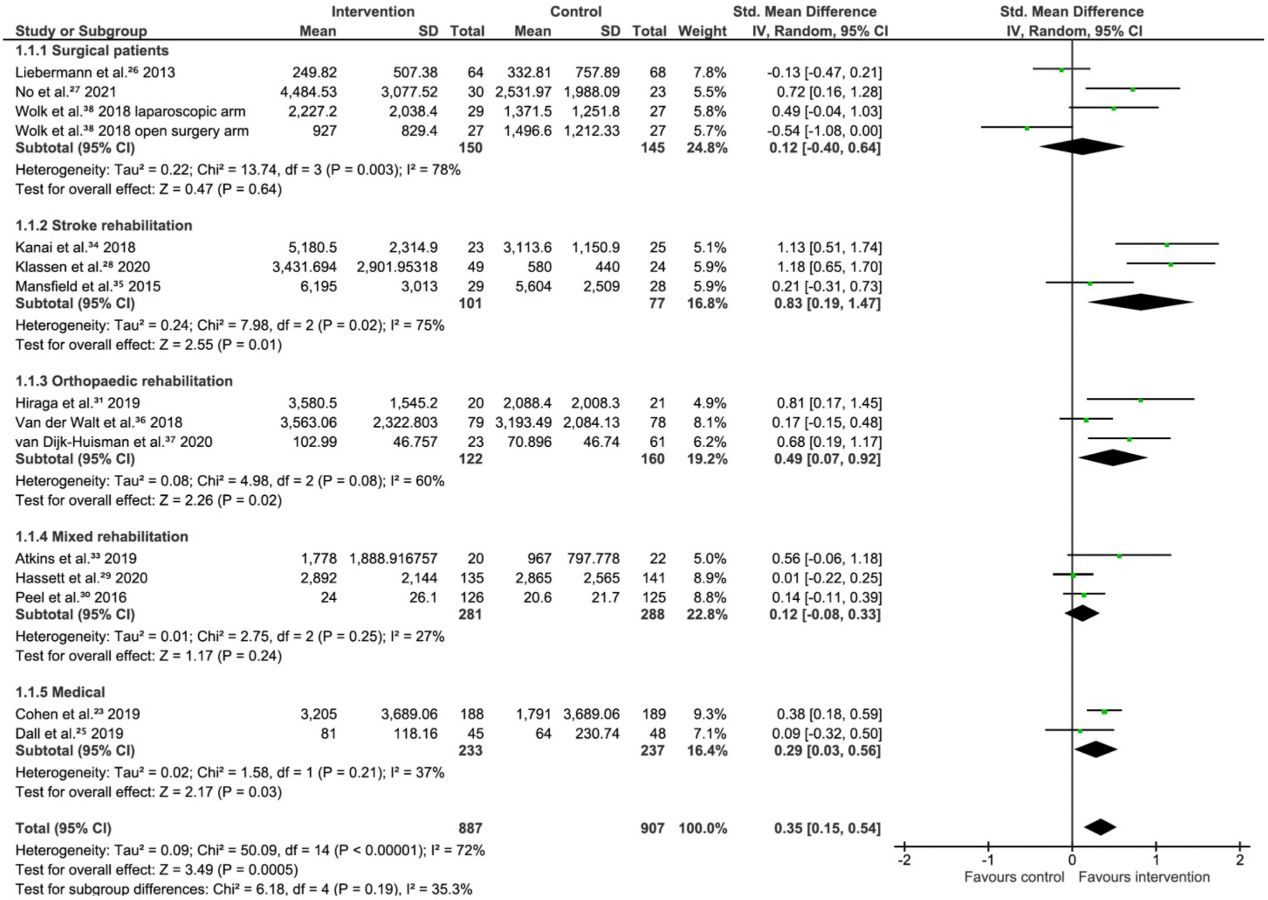

© 2023 Szeto K et al. *JAMA Network Open.*

eFigure 1.2. Subgroup Analysis for WAT Intervention Association With Length of Stay

Study or Subgroup	Intervention Mean	Intervention SD	Intervention Total	Control Mean	Control SD	Control Total	Weight	Mean Difference IV, Random, 95% CI
**1.14.1 Surgical**								
No et al.[27] 2021	12.71	13.23	30	8.48	5.7	23	3.4%	4.23 [-1.05, 9.51]
Wolk et al.[38] 2018 laparoscopic arm	10.3	4.9	29	11.3	6.5	27	8.1%	-1.00 [-4.03, 2.03]
Wolk et al.[38] 2018 open surgery arm	16.3	8	27	12.6	5.7	27	6.1%	3.70 [-0.01, 7.41]
**Subtotal (95% CI)**			**86**			**77**	**17.6%**	**1.96 [-1.60, 5.52]**
Heterogeneity: Tau² = 5.86; Chi² = 4.99, df = 2 (P = 0.08); I² = 60%								
Test for overall effect: Z = 1.08 (P = 0.28)								
**1.14.2 Stroke Rehabilitation**								
Kanai et al.[34] 2018	12.2	2.8	23	11.4	3.9	25	13.7%	0.80 [-1.11, 2.71]
Mansfield et al.[35] 2015	17.4	16.7	29	14.1	6.8	28	2.3%	3.30 [-3.28, 9.88]
**Subtotal (95% CI)**			**52**			**53**	**16.1%**	**0.99 [-0.84, 2.83]**
Heterogeneity: Tau² = 0.00; Chi² = 0.51, df = 1 (P = 0.47); I² = 0%								
Test for overall effect: Z = 1.06 (P = 0.29)								
**1.14.3 Orthopaedic rehabilitation**								
Hiraga et al.[31] 2019	31.4	4.6	20	40.8	13	21	2.8%	-9.40 [-15.31, -3.49]
van Dijk-Huisman et al.[37] 2020	3.67	1.941	27	3.87	1.668	61	21.7%	-0.20 [-1.04, 0.64]
**Subtotal (95% CI)**			**47**			**82**	**24.5%**	**-4.32 [-13.28, 4.65]**
Heterogeneity: Tau² = 37.67; Chi² = 9.11, df = 1 (P = 0.003); I² = 89%								
Test for overall effect: Z = 0.94 (P = 0.35)								
**1.14.4 Mixed rehabilitation**								
Atkins et al.[33] 2019	39	31	39	43	30	39	0.6%	-4.00 [-17.54, 9.54]
Peel et al.[30] 2016	16.3	6.9	126	15.4	7.5	125	14.6%	0.90 [-0.88, 2.68]
**Subtotal (95% CI)**			**165**			**164**	**15.2%**	**0.82 [-0.95, 2.58]**
Heterogeneity: Tau² = 0.00; Chi² = 0.49, df = 1 (P = 0.48); I² = 0%								
Test for overall effect: Z = 0.91 (P = 0.37)								
**1.14.5 Medical**								
Cohen et al.[23] 2019	5.8	3	188	6.5	4.3	189	22.3%	-0.70 [-1.45, 0.05]
Dall et al.[25] 2019	7.3	12.2	45	8.3	10.4	48	4.3%	-1.00 [-5.62, 3.62]
**Subtotal (95% CI)**			**233**			**237**	**26.6%**	**-0.71 [-1.45, 0.03]**
Heterogeneity: Tau² = 0.00; Chi² = 0.02, df = 1 (P = 0.90); I² = 0%								
Test for overall effect: Z = 1.88 (P = 0.06)								
**Total (95% CI)**			**583**			**613**	**100.0%**	**0.08 [-0.97, 1.13]**
Heterogeneity: Tau² = 1.14; Chi² = 22.24, df = 10 (P = 0.01); I² = 55%								
Test for overall effect: Z = 0.14 (P = 0.89)								
Test for subgroup differences: Chi² = 6.91, df = 4 (P = 0.14), I² = 42.1%								

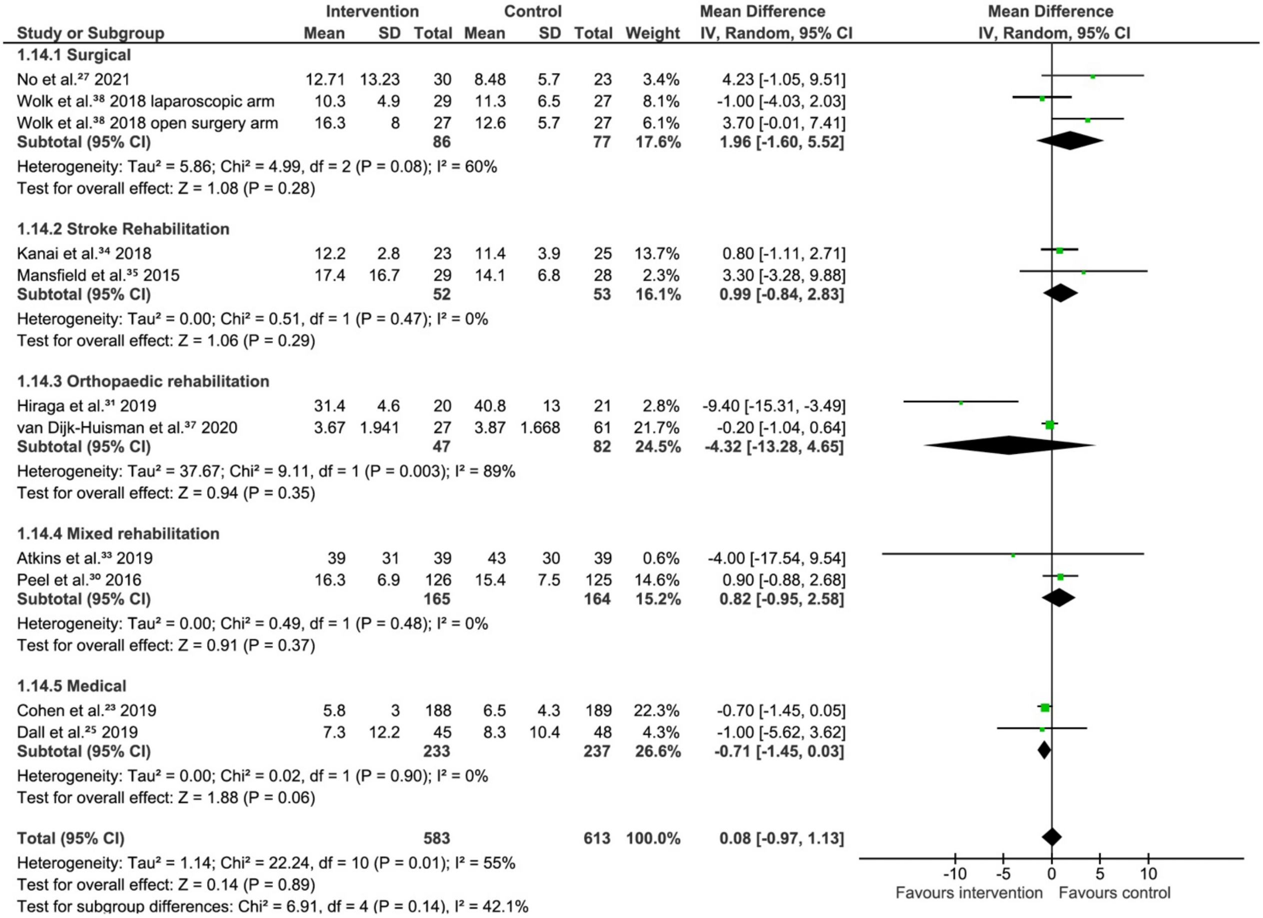

© 2023 Szeto K et al. *JAMA Network Open.*

eFigure 2. Forest Plot Meta-Analyses for Different Physical Activity Outcomes

eFigure 2.1. Forest plot of WAT intervention association with daily steps (per 1000 steps/day)

	Intervention			Control				Mean Difference
**Study or Subgroup**	**Mean**	**SD**	**Total**	**Mean**	**SD**	**Total**	**Weight**	**IV, Random, 95% CI**
Atkins et al.[33] 2019	1.778	1.888917	20	0.967	0.797778	22	7.8%	0.81 [-0.08, 1.70]
Cohen et al.[23] 2019	3.205	3.68906	188	1.791	3.68906	189	8.8%	1.41 [0.67, 2.16]
Hassett et al.[29] 2020	0.202	0.122	135	0.204	0.124	124	12.4%	-0.00 [-0.03, 0.03]
Hiraga et al.[31] 2019	3.5805	1.5452	20	2.0884	2.0084	21	6.6%	1.49 [0.40, 2.59]
Kanai et al.[34] 2018	5.1805	2.3149	23	3.1136	1.1502	25	6.8%	2.07 [1.02, 3.11]
Klassen et al.[28] 2020	3.431694	2.901953	49	0.58	0.44	24	8.2%	2.85 [2.02, 3.68]
Liebermann et al.[26] 2013	0.24982	0.50738	61	0.33281	0.75789	68	12.0%	-0.08 [-0.30, 0.14]
Mansfield et al.[35] 2015	6.195	3.013	29	5.604	2.509	28	4.9%	0.59 [-0.85, 2.03]
No et al.[27] 2021	4.48453	3.07752	30	2.53197	1.98809	23	5.2%	1.95 [0.58, 3.32]
Van der Walt et al.[36] 2018	3.56306	2.322803	79	3.19349	2.08413	78	9.2%	0.37 [-0.32, 1.06]
Wolk et al.[38] 2018 laparoscopic arm	2.2272	2.0384	29	1.3715	1.2518	27	7.9%	0.86 [-0.02, 1.74]
Wolk et al.[38] 2018 open surgery arm	0.927	0.8294	27	1.4966	1.2123	27	10.1%	-0.57 [-1.12, -0.02]
**Total (95% CI)**			**690**			**656**	**100.0%**	**0.83 [0.42, 1.24]**

Heterogeneity: Tau² = 0.35; Chi² = 101.91, df = 11 (P < 0.00001); I² = 89%
Test for overall effect: Z = 3.96 (P < 0.0001)

Mean Difference
IV, Random, 95% CI

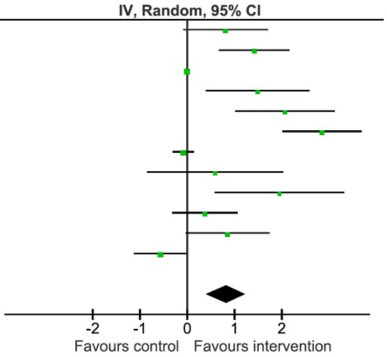

eFigure 2.2. Forest plot of WAT intervention association with active time (mins/day)

	Intervention			Control				Mean Difference
**Study or Subgroup**	**Mean**	**SD**	**Total**	**Mean**	**SD**	**Total**	**Weight**	**IV, Random, 95% CI**
Atkins et al.[33] 2019	2	1.7074465	20	2	1.79493	22	21.1%	0.00 [-1.06, 1.06]
Dall et al.[25] 2019	81	118.1627	45	64	230.74	48	1.4%	17.00 [-56.84, 90.84]
Hassett et al.[29] 2020	202	122	135	204	124	141	6.7%	-2.00 [-31.02, 27.02]
Hiraga et al.[31] 2019	29.023	9.1883	20	12.197	5.35	21	20.1%	16.83 [12.19, 21.46]
Kanai et al.[34] 2018	139.5	52	23	113.7	25.1	25	8.8%	25.80 [2.38, 49.22]
Mansfield et al.[35] 2015	75.9	34.7	29	71.5	29.7	28	12.3%	4.40 [-12.35, 21.15]
Peel et al.[30] 2016	24	26.1	126	20.6	21.7	125	19.4%	3.40 [-2.54, 9.34]
van Dijk-Huisman et al.[37] 2020	102.99	46.757	23	70.896	46.71	61	9.3%	32.09 [9.68, 54.51]
Wolk et al.[38] 2018 laparoscopic arm	482.6	286.4	29	348.3	253.6	27	0.4%	134.30 [-7.18, 275.78]
Wolk et al.[38] 2018 open surgery arm	290.3	186.8	27	462.4	315	27	0.4%	-172.10 [-310.24, -33.96]
**Total (95% CI)**			**477**			**525**	**100.0%**	**9.75 [0.65, 18.84]**

Heterogeneity: Tau² = 101.58; Chi² = 70.42, df = 9 (P < 0.00001); I² = 87%
Test for overall effect: Z = 2.10 (P = 0.04)

Mean Difference
IV, Random, 95% CI

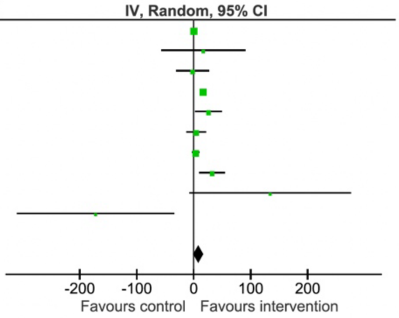

© 2023 Szeto K et al. *JAMA Network Open.*

eFigure 3. Funnel Plot Analyses

eFigure 3.1. Overall physical activity

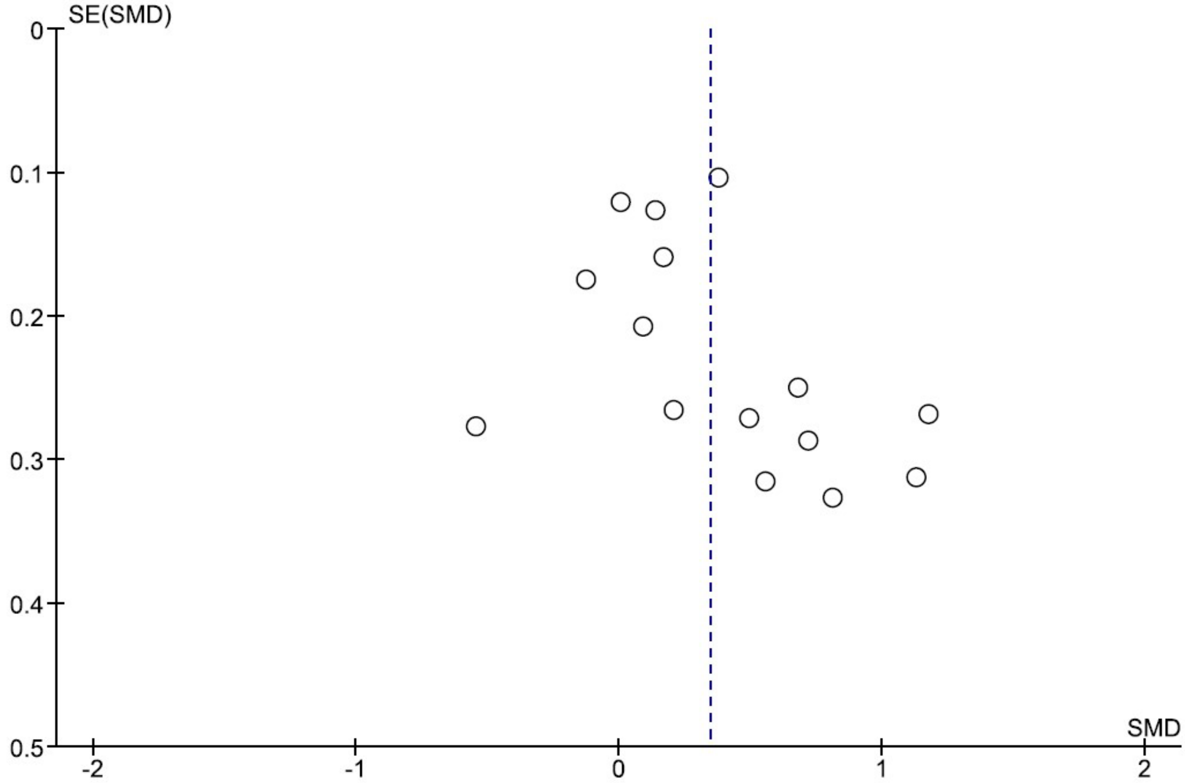

eFigure 3.2 Daily step count

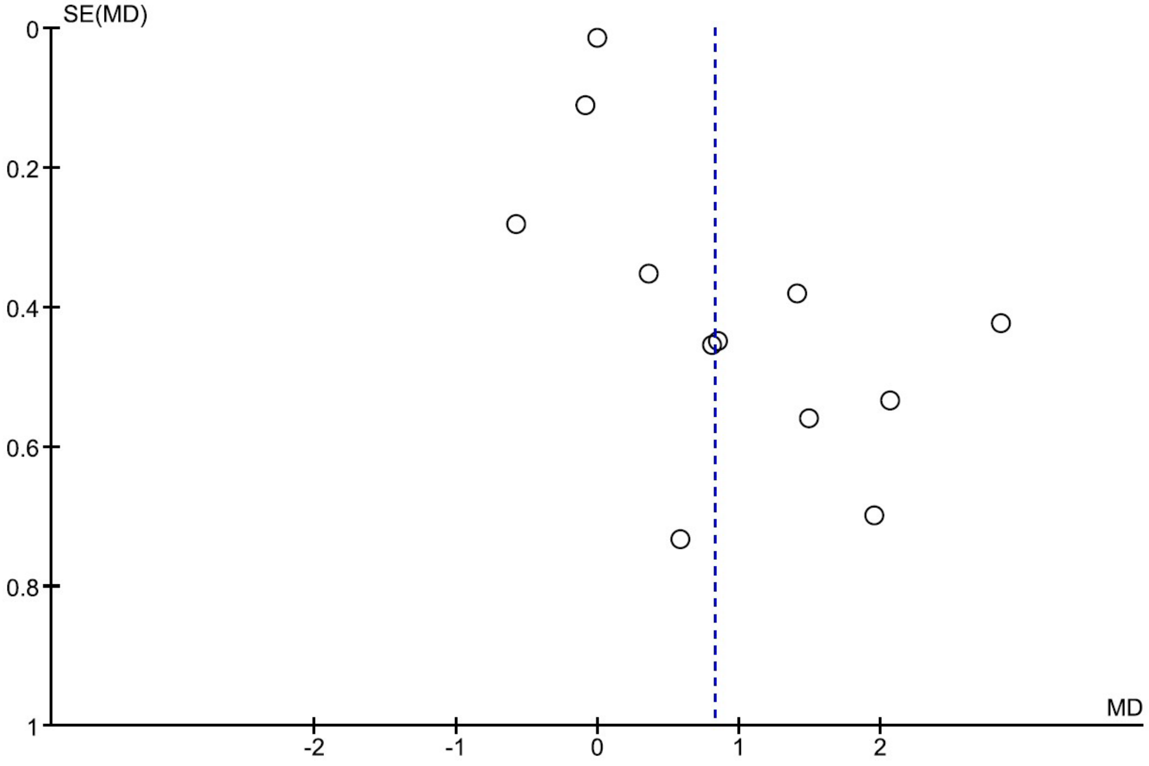

© 2023 Szeto K et al. *JAMA Network Open.*

eFigure 3.3. Length of stay

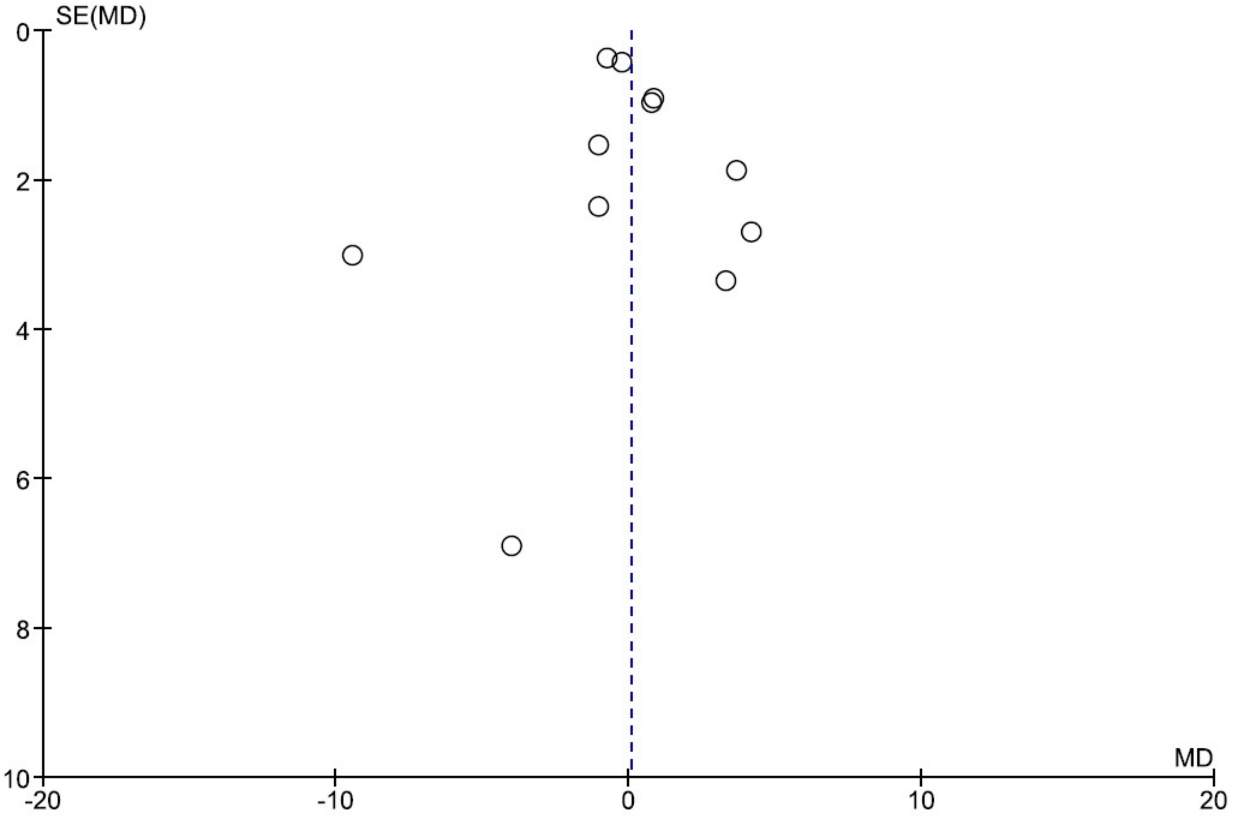

© 2023 Szeto K et al. *JAMA Network Open.*

eTable 1: Search Strategies

OVID MEDLINE
Search date: 16/03/2022
Number of results: 2416

#	Searches
**1**	patient*.mp.
**2**	(hospital* or (hospital adj3 home) or (home adj2 hospital)).mp.
**3**	exp Inpatients/
**4**	exp Patient Admission/
**5**	exp Hospitalization/
**6**	exp Hospital Medicine/
**7**	exp Home Care services/
**8**	1 or 2 or 3 or 4 or 5 or 6 or 7
**9**	((fit* or activity) adj1 (monitor* or track* or sens* or band*)).mp.
**10**	(step count* or stepcount* or ((smart or sport*) adj1 watch)).mp.
**11**	(acceleromet* or pedomet*).mp.
**12**	(Fitbit* or apple watch* or garmin* or jawbone* or polar* or activpal* or stepwatch or Samsung watch* or geneactiv* or sensewear* or actigraph* or oura* or whoop* or whitings* or xiaomi*).mp.
**13**	exp Fitness Trackers/
**14**	exp Accelerometry/
**15**	9 or 10 or 11 or 12 or 13 or 14
**16**	(trial* or intervention or RCT or nRCT or (randomi* adj2 trial) or ((nonrandomi* or non randomi*) adj2 trial) or ((quasirandomi* or quasi randomi*) adj2 trial) or pilot or feasibility).mp.
**17**	exp Randomized Controlled Trial/
**18**	16 or 17
**19**	(physical adj1 (activ* or mobil* or exercise)).mp.
**20**	(sedentary or inactiv* or (physical* adj1 (inactiv* or immobil*)) or sitting or bed rest or time in bed).mp.
**21**	(step* adj1 (count or daily)).mp.
**22**	exp Exercise/
**23**	19 or 20 or 21 or 22
**24**	8 and 15 and 18 and 23

EMBASE and EMCARE
Search date: 16/03/2022
Number of results EMBASE: 4948
Number of results EMCARE: 1444

#	Searches
**1**	patient*.mp.
**2**	(hospital* or (hospital adj3 home) or (home adj2 hospital)).mp.
**3**	exp hospital patient/
**4**	exp hospital admission/
**5**	exp Hospitalization/
**6**	hospital management/ or military hospital/ or hospital care/ or teaching hospital/ or geriatric hospital/ or general hospital/ or hospital service/ or community hospital/ or critical access hospital/ or private hospital/ or aged hospital patient/ or rural hospital/ or university hospital/ or urban hospital/ or hospital department/ or mental hospital/ or burn care hospital/ or non profit hospital/ or public hospital/ or field hospital/ or low volume hospital/
**7**	exp home care/
**8**	1 or 2 or 3 or 4 or 5 or 6 or 7
**9**	((fit* or activity) adj1 (monitor* or track* or sens* or band*)).mp.

© 2023 Szeto K et al. *JAMA Network Open.*

**10**	(step count* or stepcount* or ((smart or sport*) adj1 watch)).mp.
**11**	(acceleromet* or pedomet*).mp.
**12**	(Fitbit* or apple watch* or garmin* or jawbone* or polar* or activpal* or stepwatch or Samsung watch* or geneactiv* or sensewear* or actigraph* or oura* or whoop* or whitings* or xiaomi*).mp.
**13**	exp smart watch/
**14**	exp activity tracker/
**15**	exp accelerometer/ or exp pedometer/
**16**	9 or 10 or 11 or 12 or 13 or 14 or 15
**17**	(trial* or intervention or RCT or nRCT or (randomi* adj2 trial) or ((nonrandomi* or non randomi*) adj2 trial) or ((quasirandomi* or quasi randomi*) adj2 trial) or pilot or feasibility).mp.
**18**	exp clinical trial/ or exp randomized controlled trial/
**19**	17 or 18
**20**	(physical adj1 (activ* or mobil* or exercise)).mp.
**21**	(sedentary or inactiv* or (physical* adj1 (inactiv* or immobil*)) or sitting or bed rest or time in bed).mp.
**22**	(step* adj1 (count or daily)).mp.
**23**	exp physical activity/ or "physical activity, capacity and performance"/
**24**	20 or 21 or 22 or 23
**25**	8 and 16 and 19 and 24

CINAHL
Search date: 16/03/2022
Number of results: 1134

#	Searches
**1**	(TI (inpatient* or "in patient"* or in-patient* or patient*)) OR (AB (inpatient* or "in patient"* or in-patient* or patient*))
**2**	(TI (hospital* or (hospital n3 home) or (home n2 hospital))) OR (AB (hospital* or (hospital n3 home) or (home n2 hospital)))
**3**	(MH "Inpatients")
**4**	(MH "Patient Admission")
**5**	(MH "Hospitalization")
**6**	(MH "Hospital Medicine")
**7**	(MH "Home Health Care")
**8**	S1 OR S2 OR S3 OR S4 OR S5 OR S6 OR S7
**9**	(TI (fit* or activity) n1 (monitor* or track* or sens* or band*)) OR (AB (fit* or activity) n1 (monitor* or track* or sens* or band*))
**10**	(TI (step count* or stepcount* or ((smart or sport*) n1 watch))) OR (AB (step count* or stepcount* or ((smart or sport*) n1 watch)))
**11**	(TI (acceleromet* or pedomet*)) OR (AB (acceleromet* or pedomet*))
**12**	(TI (fitbit* or "apple watch" or garmin* or jawbone* or polar* or activpal* or stepwatch or samsung watch* or geneactiv* or sensewear* or actigraph* or oura* or whoop* or whitings* or xiaomi*)) OR (AB (fitbit* or "apple watch" or garmin* or jawbone* or polar* or activpal* or stepwatch or samsung watch* or geneactiv* or sensewear* or actigraph* or oura* or whoop* or whitings* or xiaomi*))
**13**	(MH "Wearable sensors")
**14**	(MH "Fitness trackers")
**15**	(MH "Accelerometers") OR (MH "Accelerometry") OR (MH "Pedometers")
**16**	S9 OR S10 OR S11 OR S12 OR S13 OR S14 OR S15
**17**	(TI (trial* or intervention or RCT or nRCT or (randomi* n2 trial) or ((nonrandomi* or "non randomi*" or non-randomi*) n2 trial) or ((quasirandomi* or "quasi randomi*" or quasi-randomi*) n2 trial) or pilot or feasibility)) OR (AB (trial* or intervention or RCT or nRCT or (randomi* n2 trial) or ((nonrandomi* or "non randomi*" or non-randomi*) n2 trial) or ((quasirandomi* or "quasi randomi*" or quasi-randomi*) n2 trial) or pilot or feasibility))
**18**	(MH "Experimental Studies+")
**19**	S17 OR S18

© 2023 Szeto K et al. *JAMA Network Open.*

**20**	(TI (physical n1 (activ* or mobil* or exercise)) OR (AB (physical n1 (activ* or mobil* or exercise))
**21**	(TI (sedentary or inactiv* or (physical* n1 (inactiv* or immobil*)) or sitting or bed rest or time in bed) OR (AB (sedentary or inactiv* or (physical* n1 (inactiv* or immobil*)) or sitting or bed rest or time in bed)
**22**	(TI (step* n1 (count or daily))) OR (AB (step* n1 (count or daily)))
**23**	(MH "Physical Activity")
**24**	S20 OR S21 OR S22 OR S23
**25**	S8 AND S16 AND S19 AND S24

SportsDiscuss
Search date: 16/03/2022
Number of results: 277

#	Searches
**1**	(TI (inpatient* or "in patient"* or in-patient* or patient*)) OR (AB (inpatient* or "in patient"* or in-patient* or patient*)) OR (KW (inpatient* or "in patient"* or in-patient* or patient*))
**2**	(TI (hospital* or (hospital n3 home) or (home n2 hospital))) OR (AB (hospital* or (hospital n3 home) or (home n2 hospital))) OR (KW (hospital* or (hospital n3 home) or (home n2 hospital)))
**3**	(DE "PATIENTS")
**4**	(DE "PATIENT care")
**5**	(DE "HOSPITAL CARE")
**6**	(DE "MEDICAL CARE")
**7**	S1 OR S2 OR S3 OR S4 OR S5 OR S6
**8**	(TI (fit* or activity) n1 (monitor* or track* or sens* or band*)) OR (AB (fit* or activity) n1 (monitor* or track* or sens* or band*)) OR (KW (fit* or activity) n1 (monitor* or track* or sens* or band*))
**9**	(TI (step count* or stepcount* or ((smart or sport*) n1 watch))) OR (AB (step count* or stepcount* or ((smart or sport*) n1 watch))) OR (KW (step count* or stepcount* or ((smart or sport*) n1 watch)))
**10**	(TI (acceleromet* or pedomet*)) OR (AB (acceleromet* or pedomet*)) OR (KW (acceleromet* or pedomet*))
**11**	(TI (fitbit* or "apple watch" or garmin* or jawbone* or polar* or activpal* or stepwatch or samsung watch* or geneactiv* or sensewear* or actigraph* or oura* or whoop* or whitings* or xiaomi*)) OR (AB (fitbit* or "apple watch" or garmin* or jawbone* or polar* or activpal* or stepwatch or samsung watch* or geneactiv* or sensewear* or actigraph* or oura* or whoop* or whitings* or xiaomi*)) OR (KW (fitbit* or "apple watch" or garmin* or jawbone* or polar* or activpal* or stepwatch or samsung watch* or geneactiv* or sensewear* or actigraph* or oura* or whoop* or whitings* or xiaomi*))
**12**	(DE "ACCELEROMETERS")
**13**	(DE "PEDOMETERS")
**14**	S8 OR S9 OR S10 OR S11 OR S12 OR S13
**15**	(TI (trial* or intervention or RCT or nRCT or (randomi* n2 trial) or ((nonrandomi* or "non randomi*" or non-randomi*) n2 trial) or ((quasirandomi* or "quasi randomi*" or quasi-randomi*) n2 trial) or pilot or feasibility)) OR (AB (trial* or intervention or RCT or nRCT or (randomi* n2 trial) or ((nonrandomi* or "non randomi*" or non-randomi*) n2 trial) or ((quasirandomi* or "quasi randomi*" or quasi-randomi*) n2 trial) or pilot or feasibility)) OR (KW (trial* or intervention or RCT or nRCT or (randomi* n2 trial) or ((nonrandomi* or "non randomi*" or non-randomi*) n2 trial) or ((quasirandomi* or "quasi randomi*" or quasi-randomi*) n2 trial) or pilot or feasibility))
**16**	S15
**17**	(TI (physical n1 (activ* or mobil* or exercise)) OR (AB (physical n1 (activ* or mobil* or exercise)) OR (KW (physical n1 (activ* or mobil* or exercise))
**18**	(TI (sedentary or inactiv* or (physical* n1 (inactiv* or immobil*)) or sitting or bed rest or time in bed) OR (AB (sedentary or inactiv* or (physical* n1 (inactiv* or immobil*)) or sitting or bed rest or time in bed) OR (KW (sedentary or inactiv* or (physical* n1 (inactiv* or immobil*)) or sitting or bed rest or time in bed)

© 2023 Szeto K et al. *JAMA Network Open.*

**19**	(TI (step* n1 (count or daily))) OR (AB (step* n1 (count or daily))) OR (KW (step* n1 (count or daily)))
**20**	(DE ("PHYSICAL activity" OR "PHYSICAL activity measurement"))
**21**	S17 OR S18 OR S19 OR S20
**22**	S7 AND S14 AND S16 AND S21

Scopus
Search date: 16/03/2022
Number of results: 12567

#	Searches
**1**	TITLE-ABS-KEY (patient*) or (hospital* or (hospital w/2 home) or (home w/2 hospital))
**2**	TITLE-ABS-KEY ((fit* or activity) w/2 (monitor* or track* or sens* or band*)) or (step count* or stepcount* or ((smart or sport*) w/1 watch)) or (acceleromet* or pedomet*) or (Fitbit* or apple watch* or garmin* or jawbone* or polar* or activpal* or stepwatch or Samsung watch* or geneactiv* or sensewear* or actigraph* or oura* or whoop* or whitings* or xiaomi*)
**3**	TITLE-ABS-KEY (trial* or intervention or RCT or nRCT or (randomi* w/2 trial) or ((nonrandomi* or "non randomi"*) w/2 trial) or ((quasirandomi* or "quasi randomi"*) w/2 trial) or pilot or feasibility)
**4**	TITLE-ABS-KEY (physical w/2 (activ* or mobil* or exercise)) or (sedentary or inactiv* or (physical* w/1 (inactiv* or immobil*)) or sitting or "bed rest" or "time in bed") or (step* w/1 (count or daily))
**5**	#1 AND #2 AND #3 AND #4

PEDRO
Search date: 16/03/2022
Number of results: 148

**Search 1**	
**Abstract & Title**	Inpatient physical activity
**Method**	Clinical trial
**When searching**	Match all search terms (AND)
**Search 2**	
**Abstract & Title**	Hospital at home physical activity
**Method**	Clinical trial
**When searching**	Match all search terms (AND)

© 2023 Szeto K et al. *JAMA Network Open.*

eTable 2. Risk of Bias

eTable 2.1. *Critical appraisal of randomized clinical trials included in the systematic review*

	JBI critical appraisal checklist for randomized controlled trials									
	**1. Was true randomization used for assignment of participants to treatment groups?**	**2. Was allocation to treatment groups concealed?**	**3. Were treatment groups similar at the baseline?**	**4. Were participants blind to treatment assignment?**	**5. Were those delivering treatment blind to treatment assignment?**	**6. Were outcomes assessors blind to treatment assignment?**	**7. Were treatment groups treated identically other than the intervention of interest?**	**8. Was follow up complete and if not, were differences between groups in terms of their follow up adequately described and analyzed?**	**9. Were participants analyzed in the groups to which they were randomized?**	**10. Were outcomes measured in the same way for treatment**
**Atkins et al.[1] 2019**	Yes	Yes	Yes	No	No	Yes	Yes	Yes	No	Yes
**Dall et al.[2] 2019**	No	No	Yes	No	No	Unclear	Unclear	Yes	No	Yes
**Hassett et al.[3] 2020**	Yes	Yes	Yes	No	No	Yes	Yes	Yes	Yes	Yes
**Kanai et al.[4] 2018**	Yes	Yes	Yes	Unclear	No	No	Yes	No	No	Yes
**Klassen et al.[5] 2020**	Yes	Yes	Yes	No	No	Yes	Yes	Yes	No	Yes
**Liebermann et al.[6] 2013**	Yes	Yes	Yes	Unclear	No	Unclear	Yes	Yes	No	Yes
**Mansfield et al.[7] 2015**	Yes	Yes	Yes	No	No	Yes	Yes	Yes	Unclear	Yes
**No et al.[8] 2021**	Yes	Yes	No	No	No	No	Yes	Yes	No	Yes
**Peel et al.[9] 2016**	Yes	Yes	Yes	No	No	Yes	Unclear	No	Unclear	Yes
**Van der Walt et al.[10] 2018**	Yes	Yes	Yes	No	No	Unclear	Yes	Yes	Yes	Yes

© 2023 Szeto K et al. *JAMA Network Open.*

**Wolk et al.[11] 2019**	Yes	Yes	Yes	No	No	Unclear	Yes	No	Unclear	Yes

eTable 2.2. *Critical appraisal of non-randomized clinical trials included in the systematic review*

	**JBI critical appraisal checklist for quasi-experimental trials**						
	**1. Is it clear in the study what is the 'cause' and what is the 'effect'?**	**2. Were the participants included in any comparisons similar?**	**3. Were the participants included in any comparisons receiving similar treatment/care, other than the exposure or intervention of interest?**	**4. Was there a control group?**	**5. Were there multiple measurements of the outcome both pre and post the intervention?**	**6. Was follow up complete and if not, were differences between groups in terms of their follow up adequately described and analyzed?**	**7. Were the outcomes of participants included in any comparisons measured in the same way?**
**Cohen et al.[12] 2019**	Yes	No	Unclear	Yes	Yes	Yes	Unclear
**Conijn et al.[13] 2020**	Yes	No	Yes	Yes	No	Yes	Yes
**Hiraga et al.[14] 2019**	Yes	Yes	Yes	Yes	Yes	No	Yes
**van Dijk-Huisman et al.[15] 2020**	Yes	No	Yes	Yes	No	Yes	Yes

eTable 3. Leave-1-Out Sensitivity Analyses

**Study removed**	**Result**
**Overall physical activity**	
Atkins et al. [1] 2019	(SMD=0.33; 95% CI, 0.13 to 0.54; p=0.001; I^2=74%)
Cohen et al.[12] 2019	(SMD=0.35; 95% CI, 0.13 to 0.57; p=0.002; I^2=73%)
Dall et al.[2] 2019	(SMD=0.37; 95% CI, 0.16 to 0.57; p=0.0005; I^2=74%)
Hassett et al.[3] 2020	(SMD=0.38; 95% CI, 0.17 to 0.59; p=0.0003; I^2=71%)
Hiraga et al.[14] 2019	(SMD=0.32; 95% CI, 0.12 to 0.52; p=0.001; I^2=72%)
Kanai et al.[4] 2018	(SMD=0.30; 95% CI, 0.11 to 0.49; p=0.002; I^2=69%)
Klassen et al.[5] 2020	(SMD=0.28; 95% CI, 0.11 to 0.46; p=0.002; I^2=66%)
Liebermann et al.[6] 2013	(SMD=0.38; 95% CI, 0.18 to 0.58; p=0.0002; I^2=71%)
Mansfield et al.[7] 2015	(SMD=0.36; 95% CI, 0.15 to 0.56; p=0.0006; I^2=74%)

© 2023 Szeto K et al. *JAMA Network Open.*

No et al.[8] 2021	(SMD=0.32; 95% CI, 0.12 to 0.52; p=0.001; I^2=73%)
Peel et al.[9] 2016	(SMD=0.37; 95% CI, 0.15 to 0.58; p=0.0007; I^2=73%)
Van der Walt et al.[10] 2018	(SMD=0.36; 95% CI, 0.15 to 0.58; p=0.0007; I^2=74%)
Van Dijk-Huisman et al.[15] 2020	(SMD=0.32; 95% CI, 0.12 to 0.52; p=0.0012 I^2=72%)
Wolk open surgery et al.[11] 2019	(SMD=0.39; 95% CI, 0.20 to 0.58; p<0.0001; I^2=69%)
Wolk laparoscopic et al.[11] 2019	(SMD=0.34; 95% CI, 0.13 to 0.54; p=0.001; I^2=74%)
**Daily step count**	
Atkins et al.[1] 2019	(MD=829.02; 95% CI, 400.83 to 1257.22; p=0.0001; I^2=90%)
Cohen et al.[12] 2019	(MD=756.56; 95% CI, 342.79 to 1170.33; p=0.0003; I^2=89%)
Hassett et al.[3] 2020	(MD=1015.07; 95% CI, 372.09 to 1658.05; p=0.002, I^2=89%)
Hiraga et al.[14] 2019	(MD=773.89; 95% CI, 357.45 to 1190.33; p=0.0003; I^2=89%)
Kanai et al.[4] 2018	(MD=719.19; 95% CI, 316.01 to 1122.37; p=0.0005; I^2=89%)
Klassen et al.[5] 2020	(MD=563.48; 95% CI, 218.31 to 908.65; p=0.001; I^2=82%)
Liebermann et al.[6] 2013	(MD=1016.49; 95% CI, 404.89 to 1628.1; p=0.001; I^2=90%)
Mansfield et al.[7] 2015	(MD=840.49; 95% CI, 418.11 to 1262.87; p<0.0001; I^2=90%)
No et al.[8] 2021	(MD=757.76; 95% CI, 346.94 to 1168.58; p=0.0003; I^2=89%)
Van der Walt et al.[10] 2018	(MD=879.97; 95% CI, 440.97 to 1318.97; p<0.0003; I^2=90%)
Wolk open surgery et al.[11] 2019	(MD=990.28; 95% CI, 548.92 to 1431.64; p<0.0003; I^2=90%)
Wolk laparoscopic et al.[11] 2019	(MD=824.69; 95% CI, 396.94 to 1252.44; p=0.0002; I^2=90%)
**Active time (mins/day)**	
Atkins et al.[1] 2019	(MD=12.39; 95% CI, 1.91 to 22.86; p=0.02; I^2=71%)
Dall et al.[2] 2019	(MD=9.66; 95% CI, 0.45 to 18.87; p=0.04; I^2=89%)
Hassett et al.[3] 2020	(MD=10.63; 95% CI, 1.11 to 20.16; p=0.03; I^2=89%)
Hiraga et al.[14] 2019	(MD=6.85; 95% CI, -1.43 to 15.14; p=0.11; I^2=66%)
Kanai et al.[4] 2018	(MD=8.18; 95% CI, -1.22 to 17.58; p=0.09; I^2=88%)
Mansfield et al.[7] 2015	(MD=10.57; 95% CI, 0.64 to 20.49; p=0.04; I^2=89%)
Peel et al.[9] 2016	(MD=11.58; 95% CI, -0.15 to 23.31.16; p=0.05; I^2=89%)
Van Dijk-Huisman et al.[15] 2020	(MD=7.44; 95% CI, -1.79 to 16.67; p=0.11; I^2=87%)
Wolk open surgery et al.[11] 2019	(MD=10.39; 95% CI, 1.57 to 19.22; p=0.02; I^2=88%)
Wolk laparoscopic et al.[11] 2019	(MD=9.22; 95% CI, 0.24 to 18.19; p=0.04; I^2=88%)
**Sedentary behavior (mins/day)**	
Conijn et al.[13] 2020	(MD= -17.00; 95% CI, -90.84 to 56.84; p=0.65; I^2=N/A%)
Dall et al. [2] 2019	(MD= -37.25; 95% CI, -60.27 to -14.23; p=0.002; I^2=N/A%)
**Objective measures of physical function**	
Atkins et al.[1] 2019	(SMD=0.33; 95% CI, 0.13 to 0.53; p=0.001; I^2=0%)
Hassett et al.[3] 2020	(SMD=0.30; 95% CI, 0.01 to 0.58; p=0.04; I^2=0%)
Klassen et al.[5] 2020	(SMD=0.28; 95% CI, 0.08 to 0.47; p=0.005; I^2=0%)
Mansfield et al.[7] 2015	(SMD=0.27; 95% CI, 0.08 to 0.46; p=0.006; I^2=0%)
**Mental Health**	
Hassett et al.[3] 2020	(SMD= -0.44; 95% CI, -0.91 to 0.02; p=0.06; I^2=20%)
Hiraga (A) et al.[14] 2019	(SMD= -0.01; 95% CI, -0.34 to 0.31; p=0.94; I^2=35%)
Hiraga (B) et al.[14] 2019	(SMD= -0.23; 95% CI, -0.80 to 0.33; p=0.42; I^2=75%)
Klassen et al.[5] 2020	(SMD= -0.33; 95% CI, -1.09 to 0.43; p=0.40; I^2=73%)

© 2023 Szeto K et al. *JAMA Network Open.*

**Pain**	
Hassett et al.[3] 2020	(SMD= -1.76; 95% CI, -6.34 to 2.81; p=0.45; I^2=98%)
Hiraga et al.[14] 2019	(SMD=0.19; 95% CI, -0.39 to 0.77; p=0.53; I^2=74%)
No et al.[8] 2021	(SMD= -2.05; 95% CI, -6.04 to 1.93; p=0.31; I^2=98%)
**Length of stay (days)**	
Atkins et al.[1] 2019	(MD=0.11; 95% CI, -0.97 to 1.18; p=0.85; I^2=59%)
Cohen et al.[12] 2019	(MD=0.29; 95% CI, -1.10 to 1.69; p=0.68; I^2=54%)
Dall et al.[2] 2019	(MD=0.13; 95% CI, -0.97 to 1.24; p=0.81; I^2=59%)
Hiraga et al.[14] 2019	(MD=0.13; 95% CI, -0.65 to 0.91; p=0.75; I^2=31%)
Kanai et al.[4] 2018	(MD= -0.03; 95% CI, -1.20 to 1.14; p=0.96; I^2=57%)
Mansfield et al.[7] 2015	(MD=0.00; 95% CI, -1.06 to 1.06; p=1.00; I^2=57%)
No et al.[8] 2021	(MD= -0.07; 95% CI, -1.09 to 0.95; p=0.89; I^2=54%)
Peel et al.[9] 2016	(MD= -0.06; 95% CI, -1.23 to 1.11; p=0.92; I^2=56%)
Van Dijk-Huisman et al.[15] 2020	(MD=0.17; 95% CI, -1.29 to 1.64; p=0.82; I^2=60%)
Wolk open surgery et al.[11] 2019	(MD= -0.16; 95% CI, -1.15 to 0.83; p=0.76; I^2=49%)
Wolk laparoscopic et al.[11] 2019	(MD=0.18; 95% CI, -0.95 to 1.32; p=0.75; I^2=59%)
**Risk of readmission**	
Dall et al.[2] 2019	(RR=0.74; 95% CI, 0.32 to 1.72; p=0.49; I^2=0%)
Peel et al.[9] 2016	(RR=1.31; 95% CI, 0.80 to 2.12; p=0.28; I^2=0%)
Van der Walt et al.[10] 2018	(RR=1.09; 95% CI, 0.62 to 1.91; p=0.77; I^2=31%)

© 2023 Szeto K et al. *JAMA Network Open.*

eTable 4. Reason for Exclusion of Studies Screened at Full Text

Author and year	Reason for exclusion
Agarwal et al.[16] 2016	Unsuitable study design
Ambrosio et al.[17] 2017	Unsuitable intervention
Amini et al.[18] 2021	Unsuitable intervention
Arbane et al.[19] 2014	Unsuitable intervention
Arunachalam et al.[20] 2019	Unsuitable publication type (conference abstract, thesis)
Aruncahalam et al.[21] 2018	Unsuitable publication type (conference abstract, thesis)
Ashizawa et al.[22] 2022	Unsuitable comparator/control
Ashizawa et al.[23] 2021	Unsuitable intervention
Aufwerber et al.[24] 2020	Unsuitable intervention
Aunger et al.[25] 2020	Unsuitable population (inc. setting)
Awad et al.[26] 2012	Unsuitable publication type (conference abstract, thesis)
Bade et al.[27] 2018	Unsuitable population (inc. setting)
Baker et al.[28] 2020	Unsuitable publication type (conference abstract, thesis)
Balcells Vilarnau et al.[29] 2007	Unsuitable publication type (conference abstract, thesis)
Barkley et al.[30] 2019	Unsuitable study design
Barrett et al.[31] 2021	Unsuitable population (inc. setting)
Baumann et al.[32] 2011	Unsuitable intervention
Brandes et al.[33] 2018	Outcomes of interest not available
Brauer et al.[34] 2022	Unsuitable intervention
Brouns et al.[35] 2021	Outcomes of interest not available
Campo et al.[36] 2019	Unsuitable publication type (conference abstract, thesis)
Cassidy et al.[37] 2014	Unsuitable publication type (conference abstract, thesis)
Cassidy et al.[38] 2014	Unsuitable publication type (conference abstract, thesis)
Celik Ince et al.[39] 2021	Unsuitable population (inc. setting)
Chang et al.[40] 2014	Unsuitable intervention
Cheville et al.[41] 2019	Unsuitable population (inc. setting)
Chin et al.[42] 2022	Unsuitable publication type (conference abstract, thesis)
Connell et al.[43] 2018	Unsuitable publication type (conference abstract, thesis)
Cook et al.[44] 2013	Unsuitable study design
Cowie et al.[45] 2011	Unsuitable population (inc. setting)
Creel et al.[46] 2016	Unsuitable population (inc. setting)
Cuevas-Lara et al.[47] 2020	Unsuitable publication type (conference abstract, thesis)
Cuevas-Lara et al.[48] 2022	Unsuitable intervention
Da-Silva et al.[49] 2019	Unsuitable intervention
Darabseh et al.[50] 2021	Unsuitable publication type (conference abstract, thesis)
de Blok et al.[51] 2006	Unsuitable population (inc. setting)
De La Torre Costa et al.[52] 2021	Unsuitable publication type (conference abstract, thesis)
Deenik et al.[53] 2019	Unsuitable population (inc. setting)
Deenik et al.[54] 2017	Unsuitable publication type (conference abstract, thesis)
Deenik et al.[55] 2017	Unsuitable publication type (conference abstract, thesis)
Dehghani et al.[56] 2021	Unsuitable population (inc. setting)
Dorsch et al.[57] 2015	Unsuitable comparator/control
Dorsch et al.[58] 2013	Unsuitable publication type (conference abstract, thesis)
Dorsch et al.[59] 2014	Unsuitable publication type (conference abstract, thesis)
Edgren et al.[60] 2015	Unsuitable population (inc. setting)
Feldman et al.[61] 2014	Unsuitable publication type (conference abstract, thesis)
Fiore et al.[62] 2017	Unsuitable intervention
Fleiner et al.[63] 2015	Unsuitable study design
Floegel et al.[64] 2019	Unsuitable study design
Frawley et al.[65] 2020	Unsuitable population (inc. setting)
Freene et al.[66] 2020	Unsuitable study design
Fu et al.[67] 2019	Unsuitable publication type (conference abstract, thesis)
Gabrys et al.[68] 2017	Unsuitable comparator/control
Ganer Herman et al.[69] 2020	Unsuitable comparator/control

© 2023 Szeto K et al. *JAMA Network Open.*

Garding et al.[70] 1988	Unsuitable population (inc. setting)
Geidl et al.[71] 2021	Outcomes of interest not available
Geidl et al.[72] 2017	Unsuitable publication type (conference abstract, thesis)
Graham et al.[73] 2016	Unsuitable publication type (conference abstract, thesis)
Grant et al.[74] 2018	Unsuitable publication type (conference abstract, thesis)
Hacker et al.[75] 2020	Unsuitable publication type (conference abstract, thesis)
Hakala et al.[76] 2021	Unsuitable population (inc. setting)
Hamilton et al.[77] 2019	Unsuitable intervention
Hassett et al.[78] 2019	Unsuitable publication type (conference abstract, thesis)
Henriksen et al.[79] 2002	Unsuitable intervention
Herman et al.[80] 2020	Unsuitable publication type (conference abstract, thesis)
Heron et al.[81] 2019	Unsuitable publication type (conference abstract, thesis)
Hiraga et al.[82] 2022	Outcomes of interest not available
Hiraga et al.[83] 2021	Unsuitable intervention
Hornby et al.[84] 2015	Unsuitable study design
Hornikx et al.[85] 2015	Unsuitable population (inc. setting)
Hornikx et al.[86] 2014	Unsuitable publication type (conference abstract, thesis)
Houle et al.[87] 2009	Unsuitable publication type (conference abstract, thesis)
Houle et al.[88] 2011	Unsuitable population (inc. setting)
Houle et al.[89] 2012	Unsuitable population (inc. setting)
Hubbard et al.[90] 2016	Unsuitable population (inc. setting)
Hunka et al.[91] 2011	Unsuitable publication type (conference abstract, thesis)
Hunter et al.[92] 2020	Unsuitable publication type (conference abstract, thesis)
Ifikhar et al.[93] 2022	Unsuitable publication type (conference abstract, thesis)
Izawa et al.[94] 2012	Unsuitable comparator/control
Jacot et al.[95] 2020	Unsuitable population (inc. setting)
Jarden et al.[96] 2016	Unsuitable population (inc. setting)
Jarosch et al.[97] 2020	Unsuitable intervention
Jimenez-Loaisa et al.[98] 2020	Unsuitable population (inc. setting)
Jovic et al.[99] 2017	Unsuitable publication type (conference abstract, thesis)
Kaasa et al.[100] 2015	Unsuitable publication type (conference abstract, thesis)
Kanai et al.[101] 2017	Unsuitable study design
Katogi[102] 2020	Unsuitable intervention
Kelly et al.[103] 2021	Unsuitable study design
Kern et al.[104] 2020	Unsuitable study design
Kerr et al.[105] 2017	Unsuitable study design
Khorvash et al.[106] 2020	Unsuitable population (inc. setting)
Kim et al.[107] 2014	Outcomes of interest not available
Klassen et al.[108] 2015	Unsuitable publication type (conference abstract, thesis)
Konecny et al.[109] 2021	Unsuitable publication type (conference abstract, thesis)
Ku et al.[110] 2020	Unsuitable publication type (conference abstract, thesis)
Kurebayashi et al.[111] 2021	Unsuitable intervention
Lawrie et al.[112] 2018	Outcomes of interest not available
Lee et al.[113] 2019	Unsuitable population (inc. setting)
Li et al.[114] 2020	Unsuitable intervention
Lim et al.[115] 2020	Unsuitable intervention
Lim et al.[116] 2018	Unsuitable publication type (conference abstract, thesis)
Loevezijn et al.[117] 2014	Unsuitable publication type (conference abstract, thesis)
Lorenz et al.[118] 2015	Unsuitable population (inc. setting)
Losina et al.[119] 2018	Unsuitable population (inc. setting)
Low et al.[120] 2020	Unsuitable study design
Macht et al.[121] 2016	Unsuitable publication type (conference abstract, thesis)
Magheli et al.[122] 2011	Unsuitable intervention
Mansfield et al.[123] 2014	Unsuitable publication type (conference abstract, thesis)
Martinez-Velilla et al.[124] 2021	Unsuitable intervention
Mateo[125] 2020	Unsuitable publication type (conference abstract, thesis)
Mayo et al.[126] 2015	Unsuitable publication type (conference abstract, thesis)

© 2023 Szeto K et al. *JAMA Network Open.*

Mehta et al.[127] 2020	Unsuitable population (inc. setting)
Meng et al.[128] 2016	Unsuitable intervention
Metcalf et al.[129] 2019	Unsuitable study design
Miller et al.[130] 2005	Unsuitable publication type (conference abstract, thesis)
Moore et al.[131] 2020	Unsuitable intervention
Moreno et al.[132] 2019	Unsuitable intervention
Mueller et al.[133] 2017	Unsuitable publication type (conference abstract, thesis)
Murff et al.[134] 2007	Unsuitable population (inc. setting)
Musekamp et al.[135] 2019	Unsuitable intervention
Ng et al.[136] 2021	Unsuitable study design
Ni et al.[137] 2018	Unsuitable intervention
Nishitani-Yokoyama et al.[138] 2019	Unsuitable population (inc. setting)
Nolan et al.[139] 2017	Unsuitable population (inc. setting)
Nooijen et al.[140] 2016	Unsuitable intervention
Nooijen et al.[141] 2017	Unsuitable population (inc. setting)
Nooijen et al.[142] 2016	Unsuitable population (inc. setting)
O'Neill et al.[143] 2018	Unsuitable population (inc. setting)
Oesch et al.[144] 2017	Unsuitable intervention
Orme et al.[145] 2018	Unsuitable population (inc. setting)
Ortiz-Alonso et al.[146] 2020	Unsuitable intervention
Paterson et al.[147] 2019	Unsuitable publication type (conference abstract, thesis)
Patterson et al.[148] 2020	Unsuitable publication type (conference abstract, thesis)
Paxton et al.[149] 2018	Unsuitable population (inc. setting)
Peiris et al.[150] 2012	Unsuitable intervention
Peiris et al.[151] 2012	Unsuitable publication type (conference abstract, thesis)
Pfeiffer et al.[152] 2020	Unsuitable intervention
Pol et al.[153] 2019	Outcomes of interest not available
Porserud et al.[154] 2019	Unsuitable intervention
Porserud et al.[155] 2019	Unsuitable publication type (conference abstract, thesis)
Potiaumpai et al.[156] 2021	Unsuitable intervention
Pottebaum et al.[157] 2021	Unsuitable study design
Prince et al.[158] 2018	Unsuitable population (inc. setting)
Raymond et al.[159] 2018	Unsuitable intervention
Reed et al.[160] 2021	Unsuitable intervention
Ringen et al.[161] 2018	Unsuitable study design
Rivard et al.[162] 2012	Unsuitable publication type (conference abstract, thesis)
Robinson et al.[163] 2020	Unsuitable publication type (conference abstract, thesis)
S∅rensen et al.[164] 2021	Unsuitable study design
Saez de Asteasu et al.[165] 2019	Unsuitable intervention
Said et al.[166] 2018	Unsuitable intervention
Said et al.[167] 2018	Unsuitable intervention
Said et al.[168] 2012	Unsuitable intervention
Salpakoski et al.[169] 2014	Unsuitable population (inc. setting)
Schaller et al.[170] 2016	Unsuitable intervention
Scheer et al.[171] 2017	Unsuitable study design
Schneeberger et al.[172] 2016	Unsuitable publication type (conference abstract, thesis)
Serper et al.[173] 2019	Unsuitable publication type (conference abstract, thesis)
Sharan et al.[174] 2016	Non-adult sample (<18 y.o)
Shelton et al.[175] 2009	Unsuitable population (inc. setting)
Siebens et al.[176] 2020	Unsuitable intervention
Sladkova et al.[177] 2016	Unsuitable publication type (conference abstract, thesis)
Solheim et al.[178] 2017	Unsuitable population (inc. setting)
Soto-Perez-De-Celis et al.[179] 2018	Unsuitable study design
Steele et al.[180] 2012	Unsuitable publication type (conference abstract, thesis)
Steffens et al.[181] 2021	Outcomes of interest not available
Su et al.[182] 2021	Unsuitable population (inc. setting)
Svestkova et al.[183] 2014	Unsuitable publication type (conference abstract, thesis)

© 2023 Szeto K et al. *JAMA Network Open.*

Swank et al.[184] 2020	Unsuitable intervention
Tahirah et al.[185] 2015	Unsuitable publication type (conference abstract, thesis)
Taraldsen et al.[186] 2014	Unsuitable intervention
Timmerman et al.[187] 2018	Unsuitable study design
Usui et al.[188] 2015	Unsuitable publication type (conference abstract, thesis)
Valenzuela et al.[189] 2020	Unsuitable intervention
Van Biervilet et al.[190] 2021	Unsuitable study design
Van der Peijl et al.[191] 2004	Unsuitable intervention
Vilarnau et al.[192] 2004	Unsuitable publication type (conference abstract, thesis)
Waite et al.[193] 2020	Unsuitable study design
Waller et al.[194] 2021	Unsuitable comparator/control
Waller et al.[195] 2018	Unsuitable publication type (conference abstract, thesis)
Ward et al.[196] 2021	Unsuitable study design
Waugh et al.[197] 2018	Unsuitable study design
Wedlund et al.[198] 2021	Unsuitable publication type (conference abstract, thesis)
Welsch et al.[199] 2018	Unsuitable publication type (conference abstract, thesis)
Widyastuti et al.[200] 2017	Unsuitable publication type (conference abstract, thesis)
Wiklund et al.[201] 2015	Unsuitable comparator/control
Winter et al.[202] 2011	Unsuitable publication type (conference abstract, thesis)
Wiskemann et al.[203] 2011	Unsuitable intervention
Wiskemann et al.[204] 2015	Unsuitable intervention
Wu et al.[205] 2019	Unsuitable study design
Wynter-Blyth et al.[206] 2017	Unsuitable study design
Yu et al.[207] 2022	Unsuitable intervention
Yudi et al.[208] 2017	Unsuitable publication type (conference abstract, thesis)
Zimmerman et al.[209] 2007	Unsuitable population (inc. setting)

© 2023 Szeto K et al. *JAMA Network Open.*

References

1. Atkins A, Cannell J, Barr C. Pedometers alone do not increase mobility in inpatient rehabilitation: a randomized controlled trial. *Clinical rehabilitation*. 2019;33(8):1382-1390.
2. Dall CH, Andersen H, Povlsen TM, Henriksen M. Evaluation of a technology assisted physical activity intervention among hospitalised patients: A randomised study. *European Journal of Internal Medicine*. 2019;69:50-56.
3. Hassett L, van den Berg M, Lindley RI, et al. Digitally enabled aged care and neurological rehabilitation to enhance outcomes with Activity and MObility UsiNg Technology (AMOUNT) in Australia: A randomised controlled trial. *PLoS medicine*. 2020;17(2):e1003029.
4. Kanai M, Izawa KP, Kobayashi M, et al. Effect of accelerometer-based feedback on physical activity in hospitalized patients with ischemic stroke: a randomized controlled trial. *Clinical Rehabilitation*. 2018/08/01 2018;32(8):1047-1056. doi:10.1177/0269215518755841
5. Klassen TD, Dukelow SP, Bayley MT, et al. Higher doses improve walking recovery during stroke inpatient rehabilitation. *Stroke*. 2020;51(9):2639-2648.
6. Liebermann M, Awad M, Dejong M, Rivard C, Sinacore J, Brubaker L. Ambulation of hospitalized gynecologic surgical patients: a randomized controlled trial. *Obstetrics & Gynecology*. 2013;121(3):533-537.
7. Mansfield A, Wong JS, Bryce J, et al. Use of accelerometer-based feedback of walking activity for appraising progress with walking-related goals in inpatient stroke rehabilitation: a randomized controlled trial. *Neurorehabilitation and neural repair*. 2015;29(9):847-857.
8. No JH, Kim K, Kim YB, et al. Effects of an activity tracker with feedback on physical activity in women after midline laparotomy: a randomized controlled trial. *Journal of Obstetrics and Gynaecology Research*. 2021;47(7):2544-2550.
9. Peel NM, Paul SK, Cameron ID, Crotty M, Kurrle SE, Gray LC. Promoting activity in geriatric rehabilitation: a randomized controlled trial of accelerometry. *PloS one*. 2016;11(8):e0160906.
10. Van der Walt N, Salmon LJ, Gooden B, et al. Feedback from activity trackers improves daily step count after knee and hip arthroplasty: a randomized controlled trial. *The Journal of arthroplasty*. 2018;33(11):3422-3428.
11. Wolk S, Linke S, Bogner A, et al. Use of Activity Tracking in Major Visceral Surgery—the Enhanced Perioperative Mobilization Trial: a Randomized Controlled Trial. *Journal of Gastrointestinal Surgery*. 2019/06/01 2019;23(6):1218-1226. doi:10.1007/s11605-018-3998-0
12. Cohen Y, Zisberg A, Chayat Y, et al. Walking for better outcomes and recovery: the effect of WALK-FOR in preventing hospital-associated functional decline among older adults. *The Journals of Gerontology: Series A*. 2019;74(10):1664-1670.
13. Conijn D, van Bodegom-Vos L, Volker W, et al. A multicomponent intervention to decrease sedentary time during hospitalization: a quasi-experimental pilot study. *Clinical rehabilitation*. 2020;34(7):901-915.
14. Hiraga Y, Hisano S, Nomiyama K, Hirakawa Y. Effects of using activity diary for goal setting in occupational therapy on reducing pain and improving psychological and physical performance in patients after total knee arthroplasty: A non-randomised controlled study. *Hong Kong Journal of Occupational Therapy*. 2019;32(1):53-61.
15. van Dijk-Huisman HC, Weemaes AT, Boymans TA, Lenssen AF, de Bie RA. Smartphone App with an accelerometer enhances patients' physical activity following elective orthopedic surgery: a pilot study. *Sensors*. 2020;20(15):4317.
16. Agarwal DK, Viers BR, Rivera ME, et al. Physical activity monitors can be successfully implemented in perioperative care of urology patients. Conference Abstract. *J Endourol*. 2016;30(Supplement 2):A52. doi:http://dx.doi.org/10.1089/end.2016.29020.abstracts
17. Ambrosio G, Carluccio E, Ferratini M. The NEAT-HFpEF trial. Article. *Giornale Italiano di Cardiologia*. 2017;18(2):87-91.
18. Amini R, Rajabi M, Azami H, Soltanian A. The effect of self-management intervention program on the lifestyle of postmyocardial infarction patients. Article. *Journal of Education and Health Promotion*. 2021;10(1)doi:10.4103/jehp.jehp_902_20
19. Arbane G, Douiri A, Hart N, et al. Effect of postoperative physical training on activity after curative surgery for non-small cell lung cancer: A multicentre randomised controlled trial. Article. *Physiotherapy (United Kingdom)*. 2014;100(2):100-107. doi:10.1016/j.physio.2013.12.002
20. Arunachalam D, Hale DS, Heit M. 08: Impact of postoperative activity instructions on disease-specific symptom bother and impact on activities of daily living following pelvic reconstructive surgery. Conference Abstract. *American Journal of Obstetrics and Gynecology*. March 2019;220(3 Supplement):S692-S693. doi:http://dx.doi.org/10.1016/j.ajog.2019.01.018
21. Arunachalam D, Heit M, Hale DS. Impact of postoperative activity instructions on objective measures of physical activity and recovery. Conference Abstract. *Female Pelvic Medicine and Reconstructive Surgery*. September - October 2018;24(5 Supplement 1):S35-S36. doi:http://dx.doi.org/10.1097/SPV.0000000000000617
22. Ashizawa R, Honda H, Take K, et al. Approaches to Promote Reduction in Sedentary Behavior in Patients With Minor Ischemic Stroke: A Randomized Controlled Trial. *Arch Phys Med Rehabil*. Feb 2022;103(2):255-262.e4. doi:https://dx.doi.org/10.1016/j.apmr.2021.08.019

© 2023 Szeto K et al. *JAMA Network Open.*

23. Ashizawa R, Take K, Okawara K, et al. Nonleisure-time physical activity guidance following minor ischemic stroke: A randomized clinical trial. Article. *Adapted Physical Activity Quarterly*. 2021;38(2):329-347. doi:10.1123/apaq.2020-0029
24. Aufwerber S, Heijne A, Edman G, Gravare Silbernagel K, Ackermann PW. Early mobilization does not reduce the risk of deep venous thrombosis after Achilles tendon rupture: a randomized controlled trial. Randomized Controlled Trial. *Knee Surg Sports Traumatol Arthrosc*. Jan 2020;28(1):312-319. doi:https://dx.doi.org/10.1007/s00167-019-05767-x
25. Aunger JA, Greaves CJ, Davis ET, Asamane EA, Whittaker AC, Greig CA. A novel behavioural INTErvention to REduce Sitting Time in older adults undergoing orthopaedic surgery (INTEREST): results of a randomised-controlled feasibility study. Randomized Controlled Trial. *Aging Clin Exp Res*. Dec 2020;32(12):2565-2585. doi:https://dx.doi.org/10.1007/s40520-020-01475-6
26. Awad M, Rivard C, Liebermann M, DeJong M, Sinacore J, Brubaker L. Post-operative walking enhances recovery (POWER): A randomized controlled trial. Conference Abstract. *Female Pelvic Medicine and Reconstructive Surgery*. March-April 2012;1):S4-S5. doi:http://dx.doi.org/10.1097/SPV.0b013e31824bf52d
27. Bade BC, Hyer JM, Bevill BT, et al. A Patient-Centered Activity Regimen Improves Participation in Physical Activity Interventions in Advanced-Stage Lung Cancer. Comparative Study Research Support, N.I.H., Extramural. *Integ Cancer Ther*. 09 2018;17(3):921-927. doi:https://dx.doi.org/10.1177/1534735418781739
28. Baker N, Orme M, Robinson T, Drewry S, Hagenberg A, Singh S. Does adapted cardiac rehabilitation change physical activity and sedentary behaviour for people with mild-to-moderate stroke? Conference Abstract. *Physiotherapy (United Kingdom)*. May 2020;107(Supplement 1):e98. doi:http://dx.doi.org/10.1016/j.physio.2020.03.138
29. Balcells Vilarnau E, Garcia-Aymerich J, Anto JM. [Evaluation of regular physical activity in COPD patients with an accelerometer and a questionnaire: a pilot study]. Letter. *Arch Bronconeumol*. Sep 2007;43(9):524-5. Estudio de la actividad fisica habitual mediante acelerometro y cuestionario en pacientes con EPOC.
30. Barkley R, Khalil M, Shen P, Levine EA, Votanopoulos K, Clark CJ. Feasibility of low-cost accelerometers in measuring functional recovery after major oncologic surgery. *J Surg Oncol*. Nov 28 2019;28:28. doi:https://dx.doi.org/10.1002/jso.25789
31. Barrett S, Begg S, O'Halloran P, Kingsley M. The effect of a physical activity coaching intervention on accelerometer-measured sedentary behaviours in insufficiently physically active ambulatory hospital patients. *International Journal of Environmental Research and Public Health*. 01 Jun 2021;18(11) (no pagination)5543. doi:http://dx.doi.org/10.3390/ijerph18115543
32. Baumann FT, Zopf EM, Nykamp E, et al. Physical activity for patients undergoing an allogeneic hematopoietic stem cell transplantation: benefits of a moderate exercise intervention. *European Journal of Haematology 2011 Aug;87(2):148-156*. 2011;
33. Brandes M, Wirsik N, Niehoff H, Heimsoth J, Mohring B. Impact of a tailored activity counselling intervention during inpatient rehabilitation after knee and hip arthroplasty - an explorative RCT. Randomized Controlled Trial. *BMC Musculoskelet Disord*. Jun 30 2018;19(1):209. doi:https://dx.doi.org/10.1186/s12891-018-2130-7
34. Brauer SG, Kuys SS, Ada L, Paratz JD. IMproving Physical ACtivity after stroke via Treadmill training (IMPACT) and self-management: A randomized trial. *International journal of stroke : official journal of the International Stroke Society*. 02 Mar 2022:17474930221078121. doi:https://dx.doi.org/10.1177/17474930221078121
35. Brouns B, Bodegom-Vos LV, De Kloet AJ, et al. Effect of a comprehensive erehabilitation intervention alongside conventional stroke rehabilitation on disability and healthrelated quality of life: A pre-post comparison. *J Rehabil Med*. February 2021;53 (4) (no pagination)(jrm00161)doi:https://dx.doi.org/10.2340/16501977-2812
36. Campo G, Tonet E, Chiaranda G, et al. Exercise Intervention to Improve Functional Capacity in Older Adults After Acute Coronary Syndrome. Letter. *Journal of the American College of Cardiology*. 10 December 2019;74(23):2948-2950. doi:http://dx.doi.org/10.1016/j.jacc.2019.10.010
37. Cassidy N, Byrne IA, Danaher D, Egan JJ. The irish lung fibrosis association's 2000 steps a day challenge: A pilot study to evaluate a novel home exercise programme for lung fibrosis patients. Conference Abstract. *Thorax*. December 2014;2):A133-A134. doi:http://dx.doi.org/10.1136/thoraxjnl-2014-206260.266
38. Cassidy N, Byrne I, Egan J. The 2000 steps a day challenge: A tailored exercise programme for lung fibrosis patients. Conference Abstract. *European Respiratory Journal Conference: European Respiratory Society Annual Congress*. 2014;44(SUPPL. 58)
39. Celik Ince S, Partlak Gunusen N. Effect of a nurse-led intervention program on the physical health and quality of life of individuals with severe mental illness. *Perspect Psychiatr Care*. Oct 2021;57(4):1751-1764. doi:https://dx.doi.org/10.1111/ppc.12745
40. Chang NW, Lin KC, Lee SC, Chan JYH, Lee YH, Wang KY. Effects of an early postoperative walking exercise programme on health status in lung cancer patients recovering from lung lobectomy. Article. *J Clin Nurs*. 2014;23(23-24):3391-3402. doi:10.1111/jocn.12584
41. Cheville AL, Moynihan T, Herrin J, Loprinzi C, Kroenke K. Effect of collaborative telerehabilitation on functional impairment and pain among patients with advanced-stage cancer: a randomized clinical trial [with consumer summary]. *JAMA Oncology 2019 May;5(5):644-652*. 2019;

© 2023 Szeto K et al. *JAMA Network Open.*

42. Chin M, Aaron SD. Disconcerting and Counterintuitive Findings from a Trial of Exercise in Cystic Fibrosis Can Exercise Make Our Patients Worse? Editorial. *American Journal of Respiratory and Critical Care Medicine*. 01 Feb 2022;205(3):269-270. doi:https://dx.doi.org/10.1164/rccm.202110-2446ED
43. Connell H, Van Wijck F, Granat M, Shanmugam S. Increasing physical activity and reducing sedentary behaviour in stroke survivors with the use of a personalised behavioural intervention: A feasibility study. Conference Abstract. *Int j*. December 2018;13(3 Supplement 1):60. doi:http://dx.doi.org/10.1177/1747493018801108
44. Cook DJ, Thompson JE, Prinsen SK, Dearani JA, Deschamps C. Functional Recovery in the Elderly After Major Surgery: Assessment of Mobility Recovery Using Wireless Technology. *The Annals of Thoracic Surgery*. 2013/09/01/ 2013;96(3):1057-1061. doi:https://doi.org/10.1016/j.athoracsur.2013.05.092
45. Cowie A, Thow MK, Granat MH, Mitchell SL. A comparison of home and hospital-based exercise training in heart failure: immediate and long-term effects upon physical activity level. Comparative Study Randomized Controlled Trial Research Support, Non-U.S. Gov't. *Eur J Cardiovasc Prev Rehabil*. Apr 2011;18(2):158-66. doi:https://dx.doi.org/10.1177/1741826710389389
46. Creel DB, Schuh LM, Reed CA, et al. A randomized trial comparing two interventions to increase physical activity among patients undergoing bariatric surgery. Randomized Controlled Trial. *Obesity (Silver Spring)*. 08 2016;24(8):1660-8. doi:https://dx.doi.org/10.1002/oby.21548
47. Cuevas-Lara C, Asteasu MLSD, Antonanzas-Valencia C, et al. Effects of a gamification program on mobility in hospitalized older patients: A pilot study. Conference Abstract. *European Geriatric Medicine*. December 2020;11(SUPPL 1):S139. doi:https://dx.doi.org/10.1007/s41999-020-00428-6
48. Cuevas-Lara C, De Asteasu MLS, Ramírez-Vélez R, et al. Effects of game-based interventions on functional capacity in acutely hospitalised older adults: Results of an open-label non-randomised clinical trial. Article. *Age and Ageing*. 2022;51(1)afab247. doi:10.1093/ageing/afab247
49. Da-Silva RH, Moore SA, Rodgers H, et al. Wristband Accelerometers to motiVate arm Exercises after Stroke (WAVES): a pilot randomized controlled trial. *Clin Rehabil*. 2019;33(8):1391-1403.
50. Darabseh M, Rawashdeh M, Darwish F. The effects of pedometer-based intervention on patients after total knee replacement surgeries. Conference Abstract. *Physiotherapy (United Kingdom)*. December 2021;113(Supplement 1):e35-e36. doi:https://dx.doi.org/10.1016/j.physio.2021.10.267
51. de Blok BM, de Greef MH, ten Hacken NH, Sprenger SR, Postema K, Wempe JB. The effects of a lifestyle physical activity counseling program with feedback of a pedometer during pulmonary rehabilitation in patients with COPD: a pilot study. Randomized Controlled Trial. *Patient Educ Couns*. Apr 2006;61(1):48-55.
52. De La Torre Costa J, Maier M, Rubio Ballester B, Verschure P. A combination of computer-based and wearable systems to remotely promote and monitor recovery and arm use post-stroke: Preliminary results of a randomised controlled trial. Conference Abstract. *Eur Stroke J*. 2021;6(1 SUPPL):303. doi:http://dx.doi.org/10.1177/23969873211034932
53. Deenik J, Tenback DE, Tak E, Rutters F, Hendriksen IJM, van Harten PN. Changes in physical and psychiatric health after a multidisciplinary lifestyle enhancing treatment for inpatients with severe mental illness: The MULTI study I. Research Support, Non-U.S. Gov't. *Schizophr Res*. 02 2019;204:360-367. doi:https://dx.doi.org/10.1016/j.schres.2018.07.033
54. Deenik J, Tenback D, Hendriksen I, Tak E, Van Harten P. The effects of a multidisciplinary lifestyle-enhancing treatment for severe mentally ill inpatients (MULTI-study). Conference Abstract. *European Archives of Psychiatry and Clinical Neuroscience*. September 2017;267(1 Supplement 1):S88. doi:http://dx.doi.org/10.1007/s00406-017-0824-8
55. Deenik J, Tenback D, Hendriksen I, Tak E, Van Harten P. The effect of a lifestyle intervention on physical and psychiatric health, psychosocial functioning and quality of life in long-term SMI patients. Conference Abstract. *European Archives of Psychiatry and Clinical Neuroscience*. September 2017;267(1 Supplement 1):S35. doi:http://dx.doi.org/10.1007/s00406-017-0824-8
56. Dehghani M, Cheragi M, Namdari M, Roshan VD, Dehghani M. The effectiveness of home-based cardiac rehabilitation program on cardiovascular stress indices in men and women with myocardial infarction: a randomised controlled clinical trial. Article. *Revista Colombiana de Cardiologia*. 2021;28(2):128-135. doi:10.24875/RCCAR.M21000025
57. Dorsch AK, Thomas S, Xu X, Kaiser W, Dobkin BH, investigators S. SIRRACT: An International Randomized Clinical Trial of Activity Feedback During Inpatient Stroke Rehabilitation Enabled by Wireless Sensing. Clinical Trial, Phase III Multicenter Study Randomized Controlled Trial Research Support, N.I.H., Extramural. *Neurorehabil Neural Repair*. Jun 2015;29(5):407-15. doi:https://dx.doi.org/10.1177/1545968314550369
58. Dorsch A, Thomas S, Xu C, Kaiser W, Dobkin B. Sirract: A multi-center, international, randomized clinical trial using wireless technology to affect outcomes during acute stroke rehabilitation. Conference Abstract. *Neurology Conference: 65th American Academy of Neurology Annual Meeting San Diego, CA United States Conference Publication:*. 2013;80(1 MeetingAbstracts)
59. Dorsch A, Thomas S, Xu C, Kaiser W, Dobkin B. Implementation of a multicenter, international, randomized clinical trial in subacute stroke patients using wireless health technology. Conference Abstract. *Neurorehabilitation and Neural Repair*. May 2014;28(4):NP17. doi:http://dx.doi.org/10.1177/1545968314529927

© 2023 Szeto K et al. *JAMA Network Open.*

60. Edgren J, Salpakoski A, Sihvonen SE, et al. Effects of a home-based physical rehabilitation program on physical disability after hip fracture: a randomized controlled trial. *Journal of the American Medical Directors Association 2015 Apr;16(4):350e351-350e357*. 2015;
61. Feldman DI, Martin SS, Blaha MJ. Physical activity tracking studies: The effect of an entirely mobile (mhealth) platform for data capture. Conference Abstract. *Circulation Conference: American Heart Association's*. 2014;130(SUPPL. 2)
62. Fiore JF, Jr., Castelino T, Pecorelli N, et al. Ensuring Early Mobilization Within an Enhanced Recovery Program for Colorectal Surgery: A Randomized Controlled Trial. Pragmatic Clinical Trial Randomized Controlled Trial Research Support, Non-U.S. Gov't. *Ann Surg*. 08 2017;266(2):223-231. doi:https://dx.doi.org/10.1097/SLA.0000000000002114
63. Fleiner T, Trost A, Depiereux R, Zijlstra W, Häussermann P. Geriatric psychiatry inmotion - bringing physical exercise to geriatric psychiatry: Amulti- and interdisciplinary program to promote physical activity among elderly psychiatric patients. Article. *GeroPsych: The Journal of Gerontopsychology and Geriatric Psychiatry*. 2015;28(4):173-181. doi:10.1024/1662-9647/a000135
64. Floegel TA, Allen KD, Buman MP. A pilot study examining activity monitor use in older adults with heart failure during and after hospitalization. Research Support, Non-U.S. Gov't. *Geriatr Nurs*. Mar - Apr 2019;40(2):185-189. doi:https://dx.doi.org/10.1016/j.gerinurse.2018.10.001
65. Frawley HC, Lin KY, Granger CL, Higgins R, Butler M, Denehy L. An allied health rehabilitation program for patients following surgery for abdomino-pelvic cancer: a feasibility and pilot clinical study. Article. *Support Care Cancer*. 2020;28(3):1335-1350. doi:10.1007/s00520-019-04931-w
66. Freene N, van Berlo S, McManus M, Mair T, Davey R. A Behavioral Change Smartphone App and Program (ToDo-CR) to Decrease Sedentary Behavior in Cardiac Rehabilitation Participants: Prospective Feasibility Cohort Study. *JMIR Form Res*. Nov 03 2020;4(11):e17359. doi:https://dx.doi.org/10.2196/17359
67. Fu C. Physical activity level ideal for independent functional status at discharge in intensive care patients. Conference Abstract. *American Journal of Respiratory and Critical Care Medicine Conference*. 2019;199(9)
68. Gabrys L, Sperzel S, Bernhoerster M, Banzer W, Vogt L. Real-time visual activity feedback for physical activity improvement in breast and colon cancer patients. Clinical Trial. *Res Sports Med*. Jan-Mar 2017;25(1):1-10. doi:https://dx.doi.org/10.1080/15438627.2016.1258639
69. Ganer Herman H, Kleiner I, Tairy D, et al. Effect of Digital Step Counter Feedback on Mobility After Cesarean Delivery: A Randomized Controlled Trial. *Obstetrics and gynecology*. 01 Jun 2020;135(6):1345-1352. doi:http://dx.doi.org/10.1097/AOG.0000000000003879
70. Garding BS, Kerr JC, Bay K. Effectiveness of a program of information and support for myocardial infarction patients recovering at home. *Heart & Lung 1988 Jul-Aug;17(4):355-362*. 1988;
71. Geidl W, Carl J, Schuler M, et al. Long-Term Benefits of Adding a Pedometer to Pulmonary Rehabilitation for COPD: The Randomized Controlled STAR Trial. Randomized Controlled Trial. *Int J Chron Obstruct Pulmon Dis*. 2021;16:1977-1988. doi:https://dx.doi.org/10.2147/COPD.S304976
72. Geidl W, Semrau J, Streber R, et al. Effects of a brief, pedometer-based behavioral intervention for individuals with COPD during inpatient pulmonary rehabilitation on 6-week and 6-month objectively measured physical activity: study protocol for a randomized controlled trial. Randomized Controlled Trial Research Support, Non-U.S. Gov't. *Trials*. 08 29 2017;18(1):396. doi:https://dx.doi.org/10.1186/s13063-017-2124-z
73. Graham DJ. Frontiers Commentary: The HEART Mobile Phone Trial: The Partial Mediating Effects of Self-Efficacy on Physical Activity among Cardiac Patients. Note. *Front*. 2016;466. doi:10.3389/fpubh.2016.00066
74. Grant E, Hochman J, Summapund J, et al. Engagement and outcomes among older adults with mobile health (mHealth) cardiac rehabilitation: Pilot study. Conference Abstract. *J Am Geriatr Soc*. 2018;66(Supplement 2):S284. doi:http://dx.doi.org/10.1111/jgs.15376
75. Hacker ED, Richards R, Zaid MA, Chung SY, Perkins S, Farag SS. STEPS to Enhance Physical Activity after Hematopoietic Cell Transplantation for Multiple Myeloma. Conference Abstract. *Biology of Blood and Marrow Transplantation*. March 2020;26(3 Supplement):S83. doi:http://dx.doi.org/10.1016/j.bbmt.2019.12.042
76. Hakala S, Kivisto H, Paajanen T, et al. Effectiveness of Distance Technology in Promoting Physical Activity in Cardiovascular Disease Rehabilitation: Cluster Randomized Controlled Trial, A Pilot Study. *JMIR Rehabil Assist Technol*. Jun 18 2021;8(2):e20299. doi:https://dx.doi.org/10.2196/20299
77. Hamilton AC, Lee N, Stilphen M, et al. Increasing Mobility via In-hospital Ambulation Protocol Delivered by Mobility Technicians: A Pilot Randomized Controlled Trial. *Journal of Hospital Medicine*. 2019;14(5):272-277. doi:10.12788/jhm.3153
78. Hassett L, Van Den Berg M, Weber H, et al. Patient-reported outcomes of usability and enjoyment of using digital devices in rehabilitation as part of the AMOUNT (Activity and Mobility Using Technology) randomised controlled trial. Conference Abstract. *Brain Impairment*. December 2019;20(3):340-341. doi:http://dx.doi.org/10.1017/BrImp.2019.29

© 2023 Szeto K et al. *JAMA Network Open.*

79. Henriksen MG, Jensen MB, Hansen HV, Jespersen TW, Hessov I. Enforced mobilization, early oral feeding, and balanced analgesia improve convalescence after colorectal surgery. Article. *Nutrition*. 2002;18(2):147-152. doi:10.1016/S0899-9007(01)00748-1
80. Herman HG, Kleiner I, Tairy D, et al. 623: Improving post-Cesarean mobility with personalized feedback using digital step counters - a randomized controlled trial. Conference Abstract. *American Journal of Obstetrics and Gynecology*. January 2020;222(1 Supplement):S397-S398. doi:http://dx.doi.org/10.1016/j.ajog.2019.11.639
81. Heron N, Kee F, Mant J, Cupples ME, Donnelly M. Adapted home-based cardiac rehabilitation following a TIA or minor stroke? A pilot randomised trial of "the Healthy Brain Rehabilitation Manual". Conference Abstract. *Clinical Journal of Sport Medicine*. May 2019;29(3):e54. doi:http://dx.doi.org/10.1097/JSM.0000000000000748
82. Hiraga Y, Babazono A, Hara R, Nomiyama K, Hirakawa Y. Rehabilitation interventions incorporating self-management improve psychological factors: A non-randomized controlled trial of patients after total Knee arthroplasty. Article. *Cogent Psychology*. 2022;9(1)2033468. doi:10.1080/23311908.2022.2033468
83. Hiraga Y, Hisano S, Hara R, Nomiyama K, Hirakawa Y, Hida K. Combining goal setting and achievement with occupational therapy to improve pain, psychological factors and physical activity in patients after high tibial osteotomy: A non-randomized controlled study. *Hong Kong J Occup Ther*. Jun 2021;34(1):23-29. doi:https://dx.doi.org/10.1177/1569186120985296
84. Hornby TG, Holleran CL, Leddy AL, et al. Feasibility of Focused Stepping Practice during Inpatient Rehabilitation Poststroke and Potential Contributions to Mobility Outcomes. Article. *Neurorehabilitation and Neural Repair*. 2015;29(10):923-932. doi:10.1177/1545968315572390
85. Hornikx M, Demeyer H, Camillo CA, Janssens W, Troosters T. The effects of a physical activity counseling program after an exacerbation in patients with Chronic Obstructive Pulmonary Disease: a randomized controlled pilot study. Randomized Controlled Trial Research Support, Non-U.S. Gov't. *BMC polm*. Nov 04 2015;15:136. doi:https://dx.doi.org/10.1186/s12890-015-0126-8
86. Hornikx M, Demeyer H, Camillo CAM, Janssens W, Troosters T. The effects of physical activity coaching in patients with COPD after an acute exacerbation. Conference Abstract. *European Respiratory Journal Conference: European Respiratory Society Annual Congress*. 2014;44(SUPPL. 58)
87. Houle J, Doyon O, Vadeboncoeur N, Campagna L, Diaz A, Poirier P. Compliance to physical activity and cardiovascular risk factors: A positive effect of the pedometer-based activity program led by clinical nurse specialist at 12 months follow-up. Conference Abstract. *Journal of Cardiopulmonary Rehabilitation and Prevention*. September-October 2009;29(5):333. doi:http://dx.doi.org/10.1097/01.HCR.0000361190.95525.0f
88. Houle J, Doyon O, Vadeboncoeur N, Turbide G, Diaz A, Poirier P. Innovative program to increase physical activity following an acute coronary syndrome: randomized controlled trial. Randomized Controlled Trial

Research Support, Non-U.S. Gov't. *Patient Educ Couns*. Dec 2011;85(3):e237-44. doi:https://dx.doi.org/10.1016/j.pec.2011.03.018
89. Houle J, Doyon O, Vadeboncoeur N, Turbide G, Diaz A, Poirier P. Effectiveness of a pedometer-based program using a socio-cognitive intervention on physical activity and quality of life in a setting of cardiac rehabilitation. Comparative Study Multicenter Study Randomized Controlled Trial Research Support, Non-U.S. Gov't. *Can J Cardiol*. Jan-Feb 2012;28(1):27-32. doi:https://dx.doi.org/10.1016/j.cjca.2011.09.020
90. Hubbard G, O'Carroll R, Munro J, et al. The feasibility and acceptability of trial procedures for a pragmatic randomised controlled trial of a structured physical activity intervention for people diagnosed with colorectal cancer: findings from a pilot trial of cardiac rehabilitation versus usual care (no rehabilitation) with an embedded qualitative study. *Pilot feasibility stud*. 2016;2:51.
91. Hunka N. *Pedometer use as a motivational tool for increased physical activity in bariatric surgery patients*. University of Akron; 2011. https://search.ebscohost.com/login.aspx?direct=true&AuthType=cookie,ip,shib&db=ccm&AN=109858441&site=ehost-live&scope=site&authtype=ip,shib&custid=s3684833
92. Hunter A, Hodgson L, Leckie T, et al. Socially distanced rehabilitation: A potential new normal for post-critical care recovery? Conference Abstract. *Intensive Care Medicine Experimental Conference: 33rd European Society of Intensive Care Medicine Annual Congress, ESICM*. 2020;8(SUPPL 2)doi:https://dx.doi.org/10.1186/s40635-020-00354-8
93. Ifikhar J, Condren M, Stuemky L, et al. Cystic fibrosis fitness during inpatient treatment. Conference Abstract. *Journal of Investigative Medicine*. February 2022;70(2):695. doi:https://dx.doi.org/10.1136/jim-2022-SRMC.498
94. Izawa KP, Watanabe S, Hiraki K, et al. Determination of the Effectiveness of Accelerometer Use in the Promotion of Physical Activity in Cardiac Patients: A Randomized Controlled Trial. *Archives of Physical Medicine and Rehabilitation*. 2012/11/01/ 2012;93(11):1896-1902. doi:https://doi.org/10.1016/j.apmr.2012.06.015
95. Jacot W, Arnaud A, Jarlier M, et al. Brief hospital supervision of exercise and diet during adjuvant breast cancer therapy is not enough to relieve fatigue: a multicenter randomized controlled trial. *Nutrients 2020 Oct;12(10):3081*. 2020;

© 2023 Szeto K et al. *JAMA Network Open.*

96. Jarden M, Moller T, Christensen KB, Kjeldsen L, Birgens HS, Adamsen L. Multimodal intervention integrated into the clinical management of acute leukemia improves physical function and quality of life during consolidation chemotherapy: A randomized trial 'PACE-AL'. Letter. *Haematologica*. 2016;101(7):e316-e319. doi:http://dx.doi.org/10.3324/haematol.2015.140152
97. Jarosch I, Schneeberger T, Gloeckl R, et al. Short-term effects of comprehensive pulmonary rehabilitation and its maintenance in patients with idiopathic pulmonary fibrosis: A randomized controlled trial. *J*. May 2020;9(5) (no pagination)1567. doi:http://dx.doi.org/10.3390/jcm9051567
98. Jimenez-Loaisa A, Gonzalez-Cutre D, Beltran-Carrillo VJ, Alcaraz-Ibanez M. Changes in Bariatric Patients' Physical Activity Levels and Health-Related Quality of Life Following a Postoperative Motivational Physical Activity Intervention. Research Support, Non-U.S. Gov't. *Obes Surg*. 06 2020;30(6):2302-2312. doi:https://dx.doi.org/10.1007/s11695-020-04489-1
99. Jovic E, Bird ML, Cannell J, et al. Can interactive, motion-capture-based rehabilitation in an inpatient stroke population increase physical activity levels for people undergoing rehabilitation for stroke? Conference Abstract. *Int j*. August 2017;12(3 Supplement 1):54. doi:http://dx.doi.org/10.1177/1747493017720548
100. Kaasa S, Solheim T, Laird BJA, et al. A randomised, open-label trial of a multimodal intervention (Exercise, Nutrition and Anti-inflammatory Medication) plus standard care versus standard care alone to prevent/attenuate cachexia in advanced cancer patients undergoing chemotherapy. Conference Abstract. *Journal of Clinical Oncology Conference*. 2015;33(15 SUPPL. 1)
101. Kanai M, Nozoe M, Izawa KP, et al. Promoting physical activity in hospitalized patients with mild ischemic stroke: a pilot study. Research Support, Non-U.S. Gov't. *Top*. 05 2017;24(4):256-261. doi:https://dx.doi.org/10.1080/10749357.2016.1259030
102. Katogi M. Comparison of life-behavior-promoting mobilization care with walking-only mobilization care in post-gastrointestinal surgery patients: A quasi-experimental study. *Jpn J Nurs Sci*. Oct 2020;17(4):e12348. doi:https://dx.doi.org/10.1111/jjns.12348
103. Kelly R, Jones S, Price B, Katz D, McCormick C, Pearce O. Measuring Daily Compliance With Physical Activity Tracking in Ambulatory Surgery Patients: Comparative Analysis of Five Compliance Criteria. Comparative Study Research Support, Non-U.S. Gov't. *JMIR Mhealth Uhealth*. 01 26 2021;9(1):e22846. doi:https://dx.doi.org/10.2196/22846
104. Kern L, Morvan Y, Mattar L, et al. Development and evaluation of an adapted physical activity program in anorexia nervosa inpatients: a pilot study [with consumer summary]. *European Eating Disorders Review 2020 Nov;28(6):687-700*. 2020;
105. Kerr A, Dawson J, Robertson C, Rowe P, Quinn TJ. Sit to stand activity during stroke rehabilitation. Randomized Controlled Trial Research Support, Non-U.S. Gov't. *Top*. 12 2017;24(8):562-566. doi:https://dx.doi.org/10.1080/10749357.2017.1374687
106. Khorvash F, Shahnazi H, Saadatnia M, Esteki-Ghashghaei F. Implementation of home-based health promotion program to improve flow-mediated dilation among patients with subacute stroke. *Journal of Education and Health Promotion 2020 Feb 28;9(41):Epub*. 2020;
107. Kim I, Lee H. Effects of a progressive walking program on physical activity, exercise tolerance, recovery, and post-operative complications in patients with a lung resection. Article. *Journal Korean acad*. 2014;44(4):381-390. doi:10.4040/jkan.2014.44.4.381
108. Klassen TD, Eng JJ, Bayley M, et al. Implementing an extra hour of intensive, task-specific, physical therapy daily for individuals post-stroke during inpatient rehabilitation: Feasibility data from the DOSE study. Conference Abstract. *Int j*. September 2015;4):86. doi:http://dx.doi.org/10.1111/ijs.12633-2
109. Konecny P, Vyskotova J, Gaul Alacova P, et al. Does movement affect cognitive function in patients after a stroke? Conference Abstract. *Eur J Neurol*. June 2021;28(SUPPL 1):536. doi:http://dx.doi.org/10.1111/ene.14975
110. Ku J, McPhee J, Ichter Z, et al. Fitbit activity tracking does not significantly affect outcomes after sleeve gastrectomy. Conference Abstract. *Obesity (Silver Spring)*. November 2020;28(SUPPL 2):100. doi:https://dx.doi.org/10.1002/oby.23063
111. Kurebayashi Y, Mori K, Otaki J. Effects of mild-intensity physical exercise on neurocognition in inpatients with schizophrenia: A pilot randomized controlled trial. Article. *Perspect Psychiatr Care*. 2021;doi:10.1111/ppc.12896
112. Lawrie S, Dong Y, Steins D, et al. Evaluation of a smartwatch-based intervention providing feedback of daily activity within a research-naive stroke ward: A pilot randomised controlled trial. *Pilot and Feasibility Studies*. 25 Apr 2018;4(1) (no pagination)142. doi:http://dx.doi.org/10.1186/s40814-018-0345-x
113. Lee BJ, Park YH, Lee JY, Kim SJ, Jang Y, Lee JI. Smartphone Application Versus Pedometer to Promote Physical Activity in Prostate Cancer Patients. Randomized Controlled Trial Research Support, Non-U.S. Gov't. *Telemed J E Health*. 12 2019;25(12):1231-1236. doi:https://dx.doi.org/10.1089/tmj.2018.0233
114. Li I, Bui T, Phan HT, Llado A, King C, Scrivener K. App-based supplemental exercise in rehabilitation, adherence, and effect on outcomes: a randomized controlled trial. Article. *Clin Rehabil*. 2020;34(8):1083-1093. doi:10.1177/0269215520928119

© 2023 Szeto K et al. *JAMA Network Open.*

115. Lim S, Ibrahim K, Dodds R, et al. Physical activity in hospitalised older people: the feasibility and acceptability of a volunteer-led mobility intervention in the SoMoVe TM study. Research Support, Non-U.S. Gov't. *Age Ageing*. 02 27 2020;49(2):283-291. doi:https://dx.doi.org/10.1093/ageing/afz114
116. Lim S, Ibrahim K, Dodds R, et al. The role of volunteers in preventing hospital-associated deconditioning among older people: A feasibility and acceptability study. Conference Abstract. *European Geriatric Medicine*. 2018;9(Supplement 1):S208-S209. doi:http://dx.doi.org/10.1007/s41999-018-0097-4
117. Loevezijn AA, Cameron ID, Kurrle SE, Bodegom D. Feasibility of Measuring Physical Activity Using Accelerometry in Hospitalized and Community-Living Older People with Cognitive Impairment. *J Am Geriatr Soc*. 2014;62(2):388-390. doi:10.1111/jgs.12662
118. Lorenz EC, Amer H, Dean PG, Stegall MD, Cosio FG, Cheville AL. Adherence to a pedometer-based physical activity intervention following kidney transplant and impact on metabolic parameters. Research Support, N.I.H., Extramural Research Support, Non-U.S. Gov't. *Clin Transplant*. Jun 2015;29(6):560-8. doi:https://dx.doi.org/10.1111/ctr.12553
119. Losina E, Collins JE, Deshpande BR, et al. Financial Incentives and Health Coaching to Improve Physical Activity Following Total Knee Replacement: A Randomized Controlled Trial. *Arthritis Care and Research*. May 2018;70(5):732-740. doi:http://dx.doi.org/10.1002/acr.23324
120. Low CA, Danko M, Durica KC, et al. A Real-Time Mobile Intervention to Reduce Sedentary Behavior Before and After Cancer Surgery: Usability and Feasibility Study. *JMIR Perioper Med*. Mar 23 2020;3(1):e17292. doi:https://dx.doi.org/10.2196/17292
121. Macht R, Holmstrom A, Van Orden K, et al. Utilizing activity trackers as a novel strategy to increase postoperative ambulation and evaluate the impact of mobility on bariatric surgery outcomes. Conference Abstract. *Surgical Endoscopy and Other Interventional Techniques*. March 2016;1):S478. doi:http://dx.doi.org/10.1007/s00464-016-4771-7
122. Magheli A, Knoll N, Lein M, Hinz S, Kempkensteffen C, Gralla O. Impact of fast-track postoperative care on intestinal function, pain, and length of hospital stay after laparoscopic radical prostatectomy. Randomized Controlled Trial. *J Endourol*. Jul 2011;25(7):1143-7. doi:https://dx.doi.org/10.1089/end.2011.0020
123. Mansfield A, Wong J, Inness E. Does accelerometer-based feedback increase walking activity during inpatient rehabilitation poststroke? preliminary results from a randomized controlled trial. Conference Abstract. *Neurorehabilitation and Neural Repair*. May 2014;28(4):NP19. doi:http://dx.doi.org/10.1177/1545968314529927
124. Martínez-Velilla N, Sáez De Asteasu ML, Ramírez-Vélez R, Zambom-Ferraresi F, García-Hermoso A, Izquierdo M. Recovery of the Decline in Activities of Daily Living after Hospitalization through an Individualized Exercise Program: Secondary Analysis of a Randomized Clinical Trial. Article. *Journals of Gerontology - Series A Biological Sciences and Medical Sciences*. 2021;76(8):1519-1523. doi:10.1093/gerona/glab032
125. Mateo KF. Effect of a smartphone app plus an accelerometer on physical activity and functional recovery during hospitalization after orthopedic surgery. Note. *Journal of Clinical Outcomes Management*. September 2020;27(5):202-209. doi:http://dx.doi.org/10.12788/jcom.0024
126. Mayo EN, Bakhshi Nategh B, Jacobson A, Fellows LK. Using accelerometers to monitor activation of patients in an acute stroke unit. Conference Abstract. *Int j*. September 2015;4):46-47. doi:http://dx.doi.org/10.1111/ijs.12633-2
127. Mehta SJ, Hume E, Troxel AB, et al. Effect of Remote Monitoring on Discharge to Home, Return to Activity, and Rehospitalization After Hip and Knee Arthroplasty: A Randomized Clinical Trial. Randomized Controlled Trial Research Support, N.I.H., Extramural Research Support, Non-U.S. Gov't. *JAMA netw*. 12 01 2020;3(12):e2028328. doi:https://dx.doi.org/10.1001/jamanetworkopen.2020.28328
128. Meng K, Musekamp G, Schuler M, et al. The impact of a self-management patient education program for patients with chronic heart failure undergoing inpatient cardiac rehabilitation. Article. *Patient Education and Counseling*. 2016;99(7):1190-1197. doi:10.1016/j.pec.2016.02.010
129. Metcalf M, Glazyrine V, Glavin K, et al. The Feasibility of a Health Care Application in the Treatment of Patients Undergoing Radical Cystectomy. *J Urol*. 05 2019;201(5):902-908. doi:https://dx.doi.org/10.1097/JU.0000000000000050
130. Miller CL. *A symptom management intervention in diabetic coronary artery bypass graft patients*. University of Nebraska Medical Center; 2005. https://search.ebscohost.com/login.aspx?direct=true&AuthType=cookie,ip,shib&db=ccm&AN=109846927&site=ehost-live&scope=site&authtype=ip,shib&custid=s3684833
131. Moore JL, Nordvik JE, Erichsen A, Rosseland I, Bo E, Hornby TG. Implementation of High-Intensity Stepping Training during Inpatient Stroke Rehabilitation Improves Functional Outcomes. *Stroke*. 2020:563-570. doi:http://dx.doi.org/10.1161/STROKEAHA.119.027450
132. Moreno NA, de Aquino BG, Garcia IF, et al. Physiotherapist advice to older inpatients about the importance of staying physically active during hospitalisation reduces sedentary time, increases daily steps and preserves mobility: a randomised trial. Randomized Controlled Trial Research Support, Non-U.S. Gov't. *J Physiother*. 10 2019;65(4):208-214. doi:https://dx.doi.org/10.1016/j.jphys.2019.08.006

© 2023 Szeto K et al. *JAMA Network Open.*

133. Mueller K, Kotschy-Lang N, Konig S, Wagner P. Physical activity of patients with occupational lung diseases. Conference Abstract. *European Respiratory Journal Conference: European Respiratory Society International Congress, ERS*. 2017;50(Supplement 61)doi:http://dx.doi.org/10.1183/1393003.congress-2017.PA3453
134. Murff HJ. Potential benefits of a pedometer-based walking program in an inactive patient population. Note. *Journal of Clinical Outcomes Management*. May 2007;14(5):238-244.
135. Musekamp G, Gerlich C, Ehlebracht-Konig I, et al. Evaluation of a self-management patient education programme for fibromyalgia -- results of a cluster RCT in inpatient rehabilitation. *Health Education Research 2019 Apr;34(2):209-222*. 2019;
136. Ng A, Gupta E, Bansal S, et al. Cancer Patients' Perception of Usefulness of Wearable Exercise Trackers. Research Support, Non-U.S. Gov't. *Pm R*. 08 2021;13(8):845-851. doi:https://dx.doi.org/10.1002/pmrj.12475
137. Ni CY, Wang ZH, Huang ZP, et al. Early enforced mobilization after liver resection: A prospective randomized controlled trial. Article. *International Journal of Surgery*. 2018;54:254-258. doi:10.1016/j.ijsu.2018.04.060
138. Nishitani-Yokoyama M, Miyauchi K, Shimada K, et al. Impact of physical activity on coronary plaque volume and components in acute coronary syndrome patients after early phase II cardiac rehabilitation. Article. *Circulation Journal*. 2019;83(1):101-109. doi:10.1253/circj.CJ-18-0738
139. Nolan CM, Maddocks M, Canavan JL, et al. Pedometer step count targets during pulmonary rehabilitation in chronic obstructive pulmonary disease: A randomized controlled trial. *American Journal of Respiratory and Critical Care Medicine*. 15 May 2017;195(10):1344-1352. doi:http://dx.doi.org/10.1164/rccm.201607-1372OC
140. Nooijen CFJ, Stam HJ, Schoenmakers I, et al. Working mechanisms of a behavioural intervention promoting physical activity in persons with subacute spinal cord injury. Article. *J Rehabil Med*. 2016;48(7):583-588. doi:10.2340/16501977-2110
141. Nooijen CFJ, Stam HJ, Sluis T, Valent L, Twisk J, van den Berg-Emons RJG. A behavioral intervention promoting physical activity in people with subacute spinal cord injury: secondary effects on health, social participation and quality of life [with consumer summary]. *Clinical Rehabilitation 2017 Jun;31(6):772-780*. 2017;
142. Nooijen CF, Stam HJ, Bergen MP, et al. A behavioural intervention increases physical activity in people with subacute spinal cord injury: a randomised trial. *J Physiother*. 01 Jan 2016;62(1):35-41. doi:http://dx.doi.org/10.1016/j.jphys.2015.11.003
143. O'Neill B, O'Shea O, McDonough S, et al. Clinician-Facilitated Physical Activity Intervention Versus Pulmonary Rehabilitation for Improving Physical Activity in COPD: A Feasibility Study. Comparative Study Randomized Controlled Trial Research Support, Non-U.S. Gov't. *Copd*. 06 2018;15(3):254-264. doi:https://dx.doi.org/10.1080/15412555.2018.1486396
144. Oesch P, Kool J, Fernandez-Luque L, et al. Exergames versus self-regulated exercises with instruction leaflets to improve adherence during geriatric rehabilitation: a randomized controlled trial. Randomized Controlled Trial. *BMC geriatr*. 03 23 2017;17(1):77. doi:https://dx.doi.org/10.1186/s12877-017-0467-7
145. Orme MW, Weedon AE, Saukko PM, et al. Findings of the Chronic Obstructive Pulmonary Disease-Sitting and Exacerbations Trial (COPD-SEAT) in Reducing Sedentary Time Using Wearable and Mobile Technologies With Educational Support: Randomized Controlled Feasibility Trial. *JMIR Mhealth Uhealth*. Apr 11 2018;6(4):e84. doi:https://dx.doi.org/10.2196/mhealth.9398
146. Ortiz-Alonso J, Bustamante-Ara N, Valenzuela PL, et al. Effect of a Simple Exercise Program on Hospitalization-Associated Disability in Older Patients: A Randomized Controlled Trial. Article. *J Am Med Dir Assoc*. 2020;21(4):531-537.e1. doi:10.1016/j.jamda.2019.11.027
147. Paterson S, Burridge J, Johnson L. Activity monitors to promote physical activity on an acute stroke unit-a feasibility study. Conference Abstract. *Int j*. 2019;14(4 SUPPL):46. doi:http://dx.doi.org/10.1177/1747493019882907
148. Patterson H, Beverley Z. Can developing an active ward ethos translate into cystic fibrosis patients "stepping up" during an inpatient stay? Conference Abstract. *Physiotherapy (United Kingdom)*. May 2020;107(Supplement 1):e8. doi:http://dx.doi.org/10.1016/j.physio.2020.03.014
149. Paxton RJ, Forster JE, Miller MJ, Gerron KL, Stevens-Lapsley JE, Christiansen CL. A Feasibility Study for Improved Physical Activity After Total Knee Arthroplasty. Randomized Controlled Trial Research Support, N.I.H., Extramural. *J Aging Phys Activity*. 01 01 2018;26(1):7-13. doi:https://dx.doi.org/10.1123/japa.2016-0268
150. Peiris CL, Taylor NF, Shields N. Additional Saturday allied health services increase habitual physical activity among patients receiving inpatient rehabilitation for lower limb orthopedic conditions: a randomized controlled trial. Multicenter Study Randomized Controlled Trial Research Support, Non-U.S. Gov't. *Arch Phys Med Rehabil*. Aug 2012;93(8):1365-70. doi:https://dx.doi.org/10.1016/j.apmr.2012.03.004
151. Peiris C, Shields N, Taylor NF. Extra physical therapy and occupational therapy increased physical activity levels in orthopedic rehabilitation: Randomized controlled trial. Conference Abstract. *Archives of Physical Medicine and Rehabilitation*. October 2012;93(10):E14. doi:http://dx.doi.org/10.1016/j.apmr.2012.08.032
152. Pfeiffer K, Kampe K, Klenk J, et al. Effects of an intervention to reduce fear of falling and increase physical activity during hip and pelvic fracture rehabilitation [with consumer summary]. *Age and Ageing 2020 Sep;49(5):771-778*. 2020;

© 2023 Szeto K et al. *JAMA Network Open.*

153. Pol M, Ter Riet G, Van Harting-Sveldt M, Krose B, Buurman BM. Effectiveness of sensor monitoring in a rehabilitation program for older patients after hip fracture: A three-arm stepped wedge randomized trial. Conference Abstract. *Technology and Disability*. 2019;31(Supplement 1):S121-S122. doi:http://dx.doi.org/10.3233/TAD-190005
154. Porserud A, Aly M, Nygren-Bonnier M, Hagstromer M. Objectively measured mobilisation is enhanced by a new behaviour support tool in patients undergoing abdominal cancer surgery. Controlled Clinical Trial. *Eur J Surg Oncol*. Oct 2019;45(10):1847-1853. doi:https://dx.doi.org/10.1016/j.ejso.2019.04.013
155. Porserud A, Aly M, Nygren-Bonnier M, Hagstromer M. Evaluation of enhanced mobilisation with individual goal-setting and self-monitoring after abdominal surgery due to cancer. Conference Abstract. *European Urology, Supplements*. March 2019;18(1):e1349-e1350. doi:http://dx.doi.org/10.1016/S1569-9056%2819%2930977-7
156. Potiaumpai M, Cutrono S, Medina T, et al. Multidirectional Walking in Hematopoietic Stem Cell Transplant Patients. Article. *Medicine and Science in Sports and Exercise*. 2021;53(2):258-266. doi:10.1249/MSS.0000000000002474
157. Pottebaum E, Warmoth A, Ayyappan S, et al. Wearable Monitors Facilitate Exercise in Adult and Pediatric Stem Cell Transplant. Article. *Exercise and Sport Sciences Reviews*. 2021;49(3):205-212. doi:10.1249/JES.0000000000000258
158. Prince SA, Reed JL, Cotie LM, Harris J, Pipe AL, Reid RD. Results of the Sedentary Intervention Trial in Cardiac Rehabilitation (SIT-CR Study): A pilot randomized controlled trial. Randomized Controlled Trial. *Int J Cardiol*. Oct 15 2018;269:317-324. doi:https://dx.doi.org/10.1016/j.ijcard.2018.07.082
159. Raymond MJ, Winter A, Jeffs KJ, Soh S-E, Holland AE. Acceptability of physical activity monitoring in older adults undergoing inpatient rehabilitation. *Aging Clinical & Experimental Research*. 2018;30(8):1005-1010. doi:10.1007/s40520-017-0857-x
160. Reed B, Tabone LE, Tabone JK, Szoka N, Abunnaja S, Bailey K. The use of an activity tracker to objectively measure inpatient activity after bariatric surgery. Article. *Surgery for Obesity and Related Diseases*. 2021;17(1):90-95. doi:10.1016/j.soard.2020.08.033
161. Ringen PA, Falk RS, Antonsen B, et al. Using motivational techniques to reduce cardiometabolic risk factors in long term psychiatric inpatients: A naturalistic interventional study. Article. *BMC Psychiatry*. 2018;18(1)255. doi:10.1186/s12888-018-1832-6
162. Rivard C, Liebermann M, Awad M, DeJong M, Sinacore J, Brubaker L. Post-operative walking enhancements for recovery (POWER): A randomized controlled trial. Conference Abstract. *Journal of the American College of Surgeons*. September 2012;1):S64. doi:http://dx.doi.org/10.1016/j.jamcollsurg.2012.06.178
163. Robinson SA, Goldstein RL, Cruz Rivera PN, et al. Results from a multi-site web-based physical activity intervention in COPD: Between group and site differences. Conference Abstract. *American Journal of Respiratory and Critical Care Medicine Conference: American Thoracic Society International Conference, ATS*. 2020;201(1)
164. Sørensen M, Bentzen M, Farholm A. Motivational physical activity intervention for psychiatric inpatients: A two phased singlecases experimental study. *European Journal of Adapted Physical Activity*. 2021;14(2):1-17.
165. Sáez de Asteasu ML, Martínez-Velilla N, Zambom-Ferraresi F, et al. Physical Exercise Improves Function in Acutely Hospitalized Older Patients: Secondary Analysis of a Randomized Clinical Trial. Article. *J Am Med Dir Assoc*. 2019;20(7):866-873. doi:10.1016/j.jamda.2019.04.001
166. Said CM, Morris ME, McGinley JL, et al. Additional structured physical activity does not improve walking in older people (> 60years) undergoing inpatient rehabilitation: a randomised trial [with consumer summary]. *Journal of Physiotherapy 2018 Oct;64(4):237-244*. 2018;
167. Said CM, Morris ME, McGinley JL, et al. Additional structured physical activity does not improve walking in older people (> 60 years) undergoing inpatient rehabilitation: a randomised trial. Article. *J Physiother*. 2018;64(4):237-244. doi:10.1016/j.jphys.2018.08.006
168. Said CM, Morris ME, Woodward M, Churilov L, Bernhardt J. Enhancing physical activity in older adults receiving hospital based rehabilitation: a phase II feasibility study. *BMC Geriatrics 2012 Jun 8;12(26):Epub*. 2012;
169. Salpakoski A, Tormakangas T, Edgren J, et al. Effects of a multicomponent home-based physical rehabilitation program on mobility recovery after hip fracture: a randomized controlled trial. *Journal of the American Medical Directors Association 2014 May;15(5):361-368*. 2014;
170. Schaller A, Dintsios CM, Icks A, Reibling N, Froboese I. Promoting physical activity in low back pain patients: Six months follow-up of a randomised controlled trial comparing a multicomponent intervention with a low intensity intervention. Article. *Clin Rehabil*. 2016;30(9):865-877. doi:10.1177/0269215515618730
171. Scheer JK, Bakhsheshian J, Keefe MK, et al. Initial Experience With Real-Time Continuous Physical Activity Monitoring in Patients Undergoing Spine Surgery. Multicenter Study. *Clin Spine Surg*. Dec 2017;30(10):E1434-E1443. doi:https://dx.doi.org/10.1097/BSD.0000000000000521
172. Schneeberger T, Glockl R, Jarosch I, et al. Effects of a 3-week inpatient pulmonary rehabilitation in patients with idiopathic pulmonary fbrosis-a randomized, controlled trial. Conference Abstract. *Wien Klin Wochenschr*. 2016;128(19-20):779-780. doi:http://dx.doi.org/10.1007/s00508-016-1103-9

© 2023 Szeto K et al. *JAMA Network Open.*

173. Serper M, Barankay I, Chadha S, Shults J, Reese P. A randomized, controlled, trial of interventions to promote walking after abdominal organ transplantation: The lift study. Conference Abstract. *American Journal of Transplantation*. April 2019;19(Supplement 3):533-534. doi:http://dx.doi.org/10.1111/ajt.15405
174. Sharan D, Rajkumar JS, Balakrishnan R. Efficacy of an activity monitor as a biofeedback device in cerebral palsy. *J Rehabil Assist Technol Eng*. Jan-Dec 2016;3:2055668316676032. doi:https://dx.doi.org/10.1177/2055668316676032
175. Shelton ML, Lee JQ, Morris GS, et al. A randomized control trial of a supervised versus a self-directed exercise program for allogeneic stem cell transplant patients. Article. *Psychooncology*. 2009;18(4):353-359. doi:10.1002/pon.1505
176. Siebens H, Aronow H, Edwards D, Ghasemi Z. A randomized controlled trial of exercise to improve outcomes of acute hospitalization in older adults. *Journal of the American Geriatrics Society 2000 Dec;48(12):1545-1552*. 2000;
177. Sladkova P, Svestkova O. Using of accelerometer in neurorehabilitation brain injury patients. Conference Abstract. *Brain Inj*. 2016;30(5-6):668. doi:http://dx.doi.org/10.3109/02699052.2016.1162060
178. Solheim TS, Laird BJA, Balstad TR, et al. A randomized phase II feasibility trial of a multimodal intervention for the management of cachexia in lung and pancreatic cancer. Article. *J Cachexia Sarcopenia Muscle*. 2017;8(5):778-788. doi:10.1002/jcsm.12201
179. Soto-Perez-De-Celis E, Kim H, Rojo-Castillo MP, et al. A pilot study of an accelerometer-equipped smartphone to monitor older adults with cancer receiving chemotherapy in Mexico. Research Support, Non-U.S. Gov't. *J Geriatr Oncol*. 03 2018;9(2):145-151. doi:https://dx.doi.org/10.1016/j.jgo.2017.09.008
180. Steele B, Dougherty C, Burr R, Gylys-Colwell I, Hunziker J. An intervention to enhance function in severe cardiopulmonary illness. Conference Abstract. *American Journal of Respiratory and Critical Care Medicine Conference: American Thoracic Society International Conference, ATS*. 2012;185(MeetingAbstracts)
181. Steffens D, Solomon MJ, Beckenkamp PR, et al. Individualised, targeted step count intervention following gastrointestinal cancer surgery: The Fit-4-Home randomised clinical trial. *ANZ J Surg*. Sep 22 2021;22:22. doi:https://dx.doi.org/10.1111/ans.17212
182. Su JJ, Yu DSF. Effects of a nurse-led eHealth cardiac rehabilitation programme on health outcomes of patients with coronary heart disease: A randomised controlled trial. Article. *Int J Nurs Stud*. 2021;122104040. doi:10.1016/j.ijnurstu.2021.104040
183. Svestkova O, Sladkova P, Oborna P, et al. The use of accelerometer in rehabilitation of brain damage patients with upper arm paresis. Conference Abstract. *Brain Inj*. May 2014;28(5-6):676-677. doi:http://dx.doi.org/10.3109/02699052.2014.892379
184. Swank C, Trammell M, Callender L, et al. The impact of a patient-directed activity program on functional outcomes and activity participation after stroke during inpatient rehabilitation—a randomized controlled trial. *Clin Rehabil*. 2020;34(4):504-514.
185. Tahirah F, Jenkins S, Othman SK, Ismail R, Ismail T, Hill K. A randomised controlled trial of individualised, progressed early exercise in patients hospitalised with an acute exacerbation of chronic obstructive pulmonary disease (AECOPD). Conference Abstract. *European Respiratory Journal Conference: European Respiratory Society Annual Congress*. 2015;46(SUPPL. 59)doi:http://dx.doi.org/10.1183/13993003.congress2015.PA743
186. Taraldsen K, Sletvold O, Thingstad P, et al. Physical behavior and function early after Hip Fracture surgery in patients receiving comprehensive geriatric care or orthopedic care - A randomized controlled trial. Article. *Journals of Gerontology - Series A Biological Sciences and Medical Sciences*. 2014;69 A(3):338-345. doi:10.1093/gerona/glt097
187. Timmerman JGJ, Dekker-van Weering MGHM, Wouters MWJMM, Stuiver MMM, de Kanter WW, Vollenbroek-Hutten MMRM. Physical behavior and associations with health outcomes in operable NSCLC patients: A prospective study. Article. *Lung Cancer*. 2018;119:91-98. doi:10.1016/j.lungcan.2018.03.006
188. Usui H. Visual feedback is an efficient method for increasing physical activity: The results of a randomized control trial. Conference Abstract. *Physiotherapy (United Kingdom)*. May 2015;1):eS1563-eS1564. doi:http://dx.doi.org/org/10.1016/j.physio.2015.03.1563
189. Valenzuela PL, Ortiz-Alonso J, Bustamante-Ara N, et al. Individual responsiveness to physical exercise intervention in acutely hospitalized older adults. Article. *J*. 2020;9(3)797. doi:10.3390/jcm9030797
190. Van Biervliet S, Declercq D, Dereeper S, Vermeulen D, Wurth B, De Guschtenaere A. The effect of an intensive residential rehabilitation program on body composition in patients with cystic fibrosis. *Eur J Pediatr*. June 2021;180(6):1981-1985. doi:http://dx.doi.org/10.1007/s00431-021-03943-1
191. van der Peijl ID, Vliet Vlieland TP, Versteegh MI, Lok JJ, Munneke M, Dion RA. Exercise therapy after coronary artery bypass graft surgery: a randomized comparison of a high and low frequency exercise therapy program. Clinical Trial, Comparative Study, Randomized Controlled Trial Research Support, Non-U.S. Gov't. *Ann Thorac Surg*. May 2004;77(5):1535-41.
192. Vilarnau EB, Garcia-Aymerich J, Antó JM. Evaluation of regular physical activity in COPD patients with an accelerometer and a questionnaire: A pilot study [3]. Letter. *Arch Bronconeumol*. 2007;43(9):524-525. doi:10.1157/13109477

© 2023 Szeto K et al. *JAMA Network Open.*

193. Waite I, Grant D, Mayes J, Greenwood S. Can a brief behavioural change intervention encourage hospital patients with low physical activity levels to engage and initiate a change in physical activity behaviour? Article. *Physiotherapy (United Kingdom)*. 2020;108:22-28. doi:10.1016/j.physio.2020.04.002
194. Waller GC, Kim TG, Perez S, et al. Comparing Activity Trackers With vs. Without Alarms to Increase Postoperative Ambulation: A Randomized Control Trial. Comparative Study, Randomized Controlled Trial. *Am Surg*. Jul 2021;87(7):1093-1098. doi:https://dx.doi.org/10.1177/0003134820973364
195. Waller GC, Kim T, Perez S, et al. Can a fitbit with reminder alarms motivate patients to increase postoperative ambulation? A randomized controlled trial. Conference Abstract. *Diseases of the Colon and Rectum*. May 2018;61(5):e208. doi:http://dx.doi.org/10.1097/DCR.0000000000001104
196. Ward S, Orme M, Zatloukal J, Singh S. Adherence to walking exercise prescription during pulmonary rehabilitation in COPD with a commercial activity monitor: a feasibility trial. *BMC polm*. Jan 18 2021;21(1):30. doi:https://dx.doi.org/10.1186/s12890-021-01406-9
197. Waugh A, Crumlish N, Kelleher E, Forde C, Broderick J. A feasibility study of a physiotherapy-led motivational programme to increase physical activity and improve cardiometabolic risk in people with major mental illness. Article. *Gen Hosp Psychiatry*. 2018;54:37-44. doi:10.1016/j.genhosppsych.2018.03.002
198. Wedlund L, Kvedar J. Wearables as a tool for measuring therapeutic adherence in behavioral health. Editorial. *npj digit*. December 2021;4(1) (no pagination)79. doi:http://dx.doi.org/10.1038/s41746-021-00458-9
199. Welsch T, Wolk S, Linke S, et al. Use of activity tracking in major visceral surgery-the enhanced perioperative mobilization (EPM) trial: A randomized controlled trial. Conference Abstract. *Eur Surg Res*. April 2018;59(Supplement 1):6. doi:http://dx.doi.org/10.1159/000488177
200. Widyastuti K, Makhabah DN, Rima A, Sutanto YS, Suradi S, Ambrosino N. Home based pulmonary rehabilitation with pedometers in Indonesian COPD patients. Conference Abstract. *European Respiratory Journal Conference: European Respiratory Society International Congress, ERS*. 2017;50(Supplement 61)doi:http://dx.doi.org/10.1183/1393003.congress-2017.PA777
201. Wiklund M, Sundqvist E, Fagevik Olsen M. Physical Activity in the Immediate Postoperative Phase in Patients Undergoing Roux-en-Y Gastric Bypass-a Randomized Controlled Trial. Randomized Controlled Trial Research Support, Non-U.S. Gov't. *Obes Surg*. Dec 2015;25(12):2245-50. doi:https://dx.doi.org/10.1007/s11695-015-1690-y
202. Winter C, Mueller C, Hardes J, Gosheger G, Boos J, Rosenbaum D. The impact of an intervention to increase physical activity in patients with a bone tumor. Conference Abstract. *Pediatric Blood and Cancer*. 2011;57(5):778-779. doi:http://dx.doi.org/10.1002/pbc.23299
203. Wiskemann J, Dreger P, Schwerdtfeger R, et al. Effects of a partly self-administered exercise program before, during, and after allogeneic stem cell transplantation. *Blood*. 03 Mar 2011;117(9):2604-2613. doi:http://dx.doi.org/10.1182/blood-2010-09-306308
204. Wiskemann J, Kleindienst N, Kuehl R, Dreger P, Schwerdtfeger R, Bohus M. Effects of physical exercise on survival after allogeneic stem cell transplantation [with consumer summary]. *International Journal of Cancer 2015 Dec;137(11):2749-2756*. 2015;
205. Wu JM, Ho TW, Chang YT, et al. Wearable-Based Mobile Health App in Gastric Cancer Patients for Postoperative Physical Activity Monitoring: Focus Group Study. Research Support, Non-U.S. Gov't. *JMIR Mhealth Uhealth*. 04 23 2019;7(4):e11989. doi:https://dx.doi.org/10.2196/11989
206. Wynter-Blyth V. Streamlining perioperative care for oesophago-gastric cancer surgery patients using home remote monitoring. *Primary Health Care*. 2017;27(5):27-31. doi:10.7748/phc.2017.e1225
207. Yu Z, Xie G, Qin C, Qiu X. [Study on the Benefit of Postoperative Exercise Rehabilitation in Patients with Lung Cancer Complicated with Chronic Obstructive Pulmonary Disease]. Randomized Controlled Trial. *Zhongguo Fei Ai Za Zhi*. Jan 20 2022;25(1):14-20. doi:https://dx.doi.org/10.3779/j.issn.1009-3419.2021.102.51
208. Yudi M, Clark D, Tsang D, et al. Smartphone-based, early cardiac rehabilitation in patients with acute coronary syndromes [smart-rehab trial]: A randomised controlled trial. Conference Abstract. *Heart Lung and Circulation*. 2017;26(Supplement 2):S349. doi:http://dx.doi.org/10.1016/j.hlc.2017.06.710
209. Zimmerman L, Barnason S, Schulz P, et al. The effects of a symptom management intervention on symptom evaluation, physical functioning, and physical activity for women after coronary artery bypass surgery. Article. *J Cardiovasc Nurs*. 2007;22(6):493-500. doi:10.1097/01.JCN.0000297379.06379.b6

© 2023 Szeto K et al. *JAMA Network Open.*
