# Supplementary material for: Interventions Using Wearable Activity Trackers to Improve Patient Physical Activity and Other Outcomes in Adults Who Are Hospitalized: A Systematic Review and Meta-analysis
Source: JAMA Netw Open. 2023 Jun 15;6(6):e2318478. doi: 10.1001/jamanetworkopen.2023.18478 (PMC10273021; doi:10.1001/jamanetworkopen.2023.18478)
Supplement: Supplement 2. — Data Sharing Statement [file jamanetwopen-e2318478-s002.pdf]

Data Sharing Statement

Szeto. Interventions Using Wearable Activity Trackers to Improve Patient Physical Activity and Other Outcomes in Adults Who Are Hospitalized. *JAMA Netw Open*. Published June 15, 2023. doi:10.1001/jamanetworkopen.2023.18478

Data
Data available: No

Additional Information
Explanation for why data not available: All data for this systematic review and meta-analysis were obtained from published trials.
